# The hologenome of *Daphnia magna* reveals possible DNA methylation and microbiome-mediated evolution of the host genome

**DOI:** 10.1093/nar/gkad685

**Published:** 2023-08-28

**Authors:** Anurag Chaturvedi, Xiaojing Li, Vignesh Dhandapani, Hollie Marshall, Stephen Kissane, Maria Cuenca-Cambronero, Giovanni Asole, Ferriol Calvet, Marina Ruiz-Romero, Paolo Marangio, Roderic Guigó, Daria Rago, Leda Mirbahai, Niamh Eastwood, John K Colbourne, Jiarui Zhou, Eamonn Mallon, Luisa Orsini

**Affiliations:** Environmental Genomics Group, School of Biosciences, and Institute for Interdisciplinary Data Science and AI, the University of Birmingham, Birmingham B15 2TT, UK; Environmental Genomics Group, School of Biosciences, and Institute for Interdisciplinary Data Science and AI, the University of Birmingham, Birmingham B15 2TT, UK; Environmental Genomics Group, School of Biosciences, and Institute for Interdisciplinary Data Science and AI, the University of Birmingham, Birmingham B15 2TT, UK; Environmental Genomics Group, School of Biosciences, and Institute for Interdisciplinary Data Science and AI, the University of Birmingham, Birmingham B15 2TT, UK; Department of Genetics and Genome Biology, the University of Leicester, Leicester LE1 7RH, UK; Environmental Genomics Group, School of Biosciences, and Institute for Interdisciplinary Data Science and AI, the University of Birmingham, Birmingham B15 2TT, UK; Environmental Genomics Group, School of Biosciences, and Institute for Interdisciplinary Data Science and AI, the University of Birmingham, Birmingham B15 2TT, UK; Aquatic Ecology Group, University of Vic - Central University of Catalonia, 08500 Vic, Spain; Centre for Genomic Regulation (CRG), The Barcelona Institute for Science and Technology (BIST), Barcelona, Catalonia, Spain; Centre for Genomic Regulation (CRG), The Barcelona Institute for Science and Technology (BIST), Barcelona, Catalonia, Spain; Centre for Genomic Regulation (CRG), The Barcelona Institute for Science and Technology (BIST), Barcelona, Catalonia, Spain; Centre for Genomic Regulation (CRG), The Barcelona Institute for Science and Technology (BIST), Barcelona, Catalonia, Spain; Centre for Genomic Regulation (CRG), The Barcelona Institute for Science and Technology (BIST), Barcelona, Catalonia, Spain; Environmental Genomics Group, School of Biosciences, and Institute for Interdisciplinary Data Science and AI, the University of Birmingham, Birmingham B15 2TT, UK; Warwick Medical School, University of Warwick, Coventry CV4 7AL, UK; Environmental Genomics Group, School of Biosciences, and Institute for Interdisciplinary Data Science and AI, the University of Birmingham, Birmingham B15 2TT, UK; Environmental Genomics Group, School of Biosciences, and Institute for Interdisciplinary Data Science and AI, the University of Birmingham, Birmingham B15 2TT, UK; Environmental Genomics Group, School of Biosciences, and Institute for Interdisciplinary Data Science and AI, the University of Birmingham, Birmingham B15 2TT, UK; Department of Genetics and Genome Biology, the University of Leicester, Leicester LE1 7RH, UK; Environmental Genomics Group, School of Biosciences, and Institute for Interdisciplinary Data Science and AI, the University of Birmingham, Birmingham B15 2TT, UK; The Alan Turing Institute, British Library, London NW1 2DB, UK

## Abstract

Properties that make organisms ideal laboratory models in developmental and medical research are often the ones that also make them less representative of wild relatives. The waterflea *Daphnia magna* is an exception, by both sharing many properties with established laboratory models and being a keystone species, a sentinel species for assessing water quality, an indicator of environmental change and an established ecotoxicology model. Yet, *Daphnia*’s full potential has not been fully exploited because of the challenges associated with assembling and annotating its gene-rich genome. Here, we present the first hologenome of *Daphnia magna*, consisting of a chromosomal-level assembly of the *D. magna* genome and the draft assembly of its metagenome. By sequencing and mapping transcriptomes from exposures to environmental conditions and from developmental morphological landmarks, we expand the previously annotates gene set for this species. We also provide evidence for the potential role of gene-body DNA-methylation as a mutagen mediating genome evolution. For the first time, our study shows that the gut microbes provide resistance to commonly used antibiotics and virulence factors, potentially mediating *Daphnia's* environmental-driven rapid evolution. Key findings in this study improve our understanding of the contribution of DNA methylation and gut microbiota to genome evolution in response to rapidly changing environments.

## Graphical Abstract

**Graphical Abstract:**
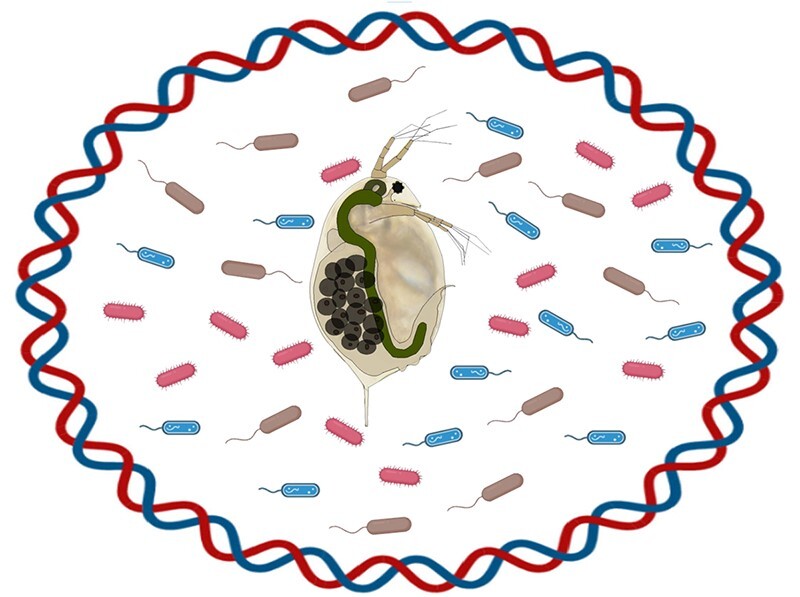

## INTRODUCTION

Model organisms possess particular traits that elevated them to their role of models: *Drosophila melanogaster* and *Danio rerio* are established models for developmental and medical research (1–4); *Escherichia coli* is used in molecular genetics (5); *Arabidopsis thaliana* is used in genome evolution and speciation (6,7); *Caenorhabitis elegans* is the organism of choice for human disease pathology (8). Many of these organisms are surrogate species for human health and are often preferred over vertebrate models (e.g. mouse) because of their versatility, ease of handling, high fecundity, and external fertilization (9). They also have extensive genetic resources, and typically have small genomes that facilitate genetic manipulation and genome-wide studies. However, they significantly differ from their wild relatives for a variety of reasons: some species are domesticated, others are human commensals or have been selected for their properties but do not share common traits with close relatives in the wild (9).

Elevating species that occupy a central role in natural ecosystems to the role of models enables studies of processes occurring in natural populations that vary over time and environmental conditions including speciation (10), the role of epigenetics in adaptation (11), acclimation and genome evolution (12–16). With the higher accessibility of high throughput sequence methodologies and the lower requirements for input material, omics profiling of ecologically relevant species is no longer a bottleneck (17,18). However, interpreting these omics profiles using reference databases is still a challenge, because existing databases have an overrepresentation of functional categories only partially relevant in ecological contexts (e.g. (19,20)). To partially alleviate these limitations, coordination among genes can be used to identify co-expression networks, in which highly correlated features form co-response modules. These co-response modules can be mapped onto functional pathways and genes of unknown function associated with genes of known function, greatly improving our understanding of their links to ecologically relevant functions (21–24).

*Daphnia* are keystone branchipod crustaceans (order Cladocera) in freshwater ecosystems worldwide (25,26). They have a wide geographic distribution (27) and occur in diverse ecological habitats ranging from fresh to brackish waters (28). They play a central role in aquatic food webs, where they are the preferred food for small vertebrate and invertebrate predators, and active grazers of algae and bacteria (26). *Daphnia magna* is an established model species in ecology, evolution, and ecotoxicology (21,25). It is used to set regulatory limits on hazardous substances in the environment using New Approach Methodologies (NAMs) for environmental and human safety assessment (29). *Daphnia's* responsiveness to natural and anthropogenic stressors is documented through extensive experimental data (e.g. (30–33)). Since the early 2010s, the role of *D. magna* in genetics and genomics has been increasingly appreciated because of the many advantages that this crustacean shares with other biomedical model organisms: short generation time that enables experimental manipulation of large populations (26); a parthenogenetic life cycle that allows the rearing of populations of identical clones from single genotypes, providing a unique system to concurrently study omics and fitness responses to environmental changes while controlling for genetic variation (25); and an exceptionally long dormancy that enables to revive temporal populations across gradients of selections spanning hundreds of years (34,35). Despite these many advantages, genomic studies have been scarce (e.g. (12,36)), largely because of the challenges associated with assembling its gene-rich genome and the high number of lineage-specific and duplicated genes, which have been documented in other *Daphnia* species, e.g. (37) and are not yet functionally annotated.

Here, we provide the first chromosomal-level genome assembly of *D. magna*, together with an updated gene set complementing existing gene annotations in this species with novel genes, supported by transcriptome data obtained from environmental exposures and developmental morphological landmarks. To understand the *Daphnia* functional landscape, we investigated DNA methylation's role in gene function evolution and the role of the microbiome in modulating *Daphnia's* genome evolution. The characterization of the *D. magna* holobiome reveals important insights into modes of adaptation elevating this species to a premier model in ecological genomics.

## MATERIALS AND METHODS

### Source material

*Daphnia magna* dormant embryos were previously resurrected (revived) from a biological archive of Lake Ring, a shallow lake in Denmark (55^0^ 57′ 51.83′′ N, 9°35′46.87′′E) (30,38). Historical records show the abundant presence of *D. magna* throughout the last century in this lake (30). Several isolates were hatched from the sedimentary archive and maintained as clonal lines in standard laboratory conditions for several generations (16:8-h light: dark regime, 10°C and fed 0.4 mg carbon/l of *Chlorella vulgaris* biweekly). One of the isolates resurrected from the modern sediment layers (LRV0_1) was used in this study to generate the first chromosomal-level genome assembly of *D. magna*, and its associated metagenome. The same genotype was used to generate RNA Seq, methylation and ATAC Seq data.

### De novo hybrid genome assembly

The *D. magna* genotype LRV0_1 was used to generate long reads with Oxford Nanopore Technologies (ONT), short reads with Illumina technologies and proximity ligation sequences with Hi-C technology (Supplementary Figure S1). The genotype was cultured in standard laboratory conditions (16:8 light: dark regime, 20°C and 0.8 mg C/l of *Chlorella vulgaris* daily) to generate sufficient material for the extraction of high molecular weight genomic DNA (gDNA). Two days before tissue collection for the ONT and the Illumina sequencing, the cultures were treated with a cocktail of antibiotics at a final concentration of 20 mg/l (Tetracycline-T, Streptomycin-S, Ampicillin-A) to reduce bacterial contamination from gut microbes in downstream analyses. The clonal replicates used in the proximity ligation experiment were not treated with antibiotics to enable the sequencing of both the host DNA and its metagenome (see below). Prior to tissue collection for gDNA extraction, *Daphnia* cultures were deprived of food for 48 h to reduce contamination from the feedstock (algae) in downstream analyses. Genomic DNA was extracted for Nanopore and Illumina libraries using the Masterpure DNA purification kit (Lucigen). Flash frozen tissue was shipped to Phase Genomics Seattle to generate Hi-C libraries, whereas ONT and Illumina compatible libraries were prepared by EnviSion (https://www.envision-service.com/).

#### Long reads sequencing (oxford nanopore technology)

Two micrograms or more of the high molecular weight DNA size—selected on Sage BluePippin gels (Sage Science, Beverly, MA, USA) were used as the starting material for the preparation of three ONT libraries (insert size: 8, 15 and 48 kb), following the manufacturer's guidelines for the Ligation Sequencing SQK-LSK108 1D kit (Oxford Nanopore Technologies, Cambridge UK). The three libraries were sequenced on R9.4.1 flow cells on the MinION sequencing device. The ONT MinKNOW Software (Oxford Nanopore Technologies, Cambridge UK) was used for base-calling using the ‘high accuracy’ configuration (dna_r9.4.1_450bps_hac.cfg) and the high-accuracy flip-flop model with config ‘dna_r9.4.1_450bps_hac.cfg.

#### Short reads sequencing (illumina technology)

Up to 1 μg of gDNA was sheared using a Bioruptor® Pico ultrasonicator with integrated cooling module (Diagenode — B01060010), following cooling on ice for 10 min. Sheared gDNA was assayed on a 2200 TapeStation (Agilent) with High Sensitivity DNA screen tapes. The sheared gDNA was then prepared into Illumina compatible DNA 250 bp paired-end libraries using KAPA HyperPrep Kit (Roche—KK8504), without amplification. Following library construction, libraries were assessed for quality and quantified on a 2200 TapeStation (Agilent) with High Sensitivity DNA Screen tapes; the libraries were sequenced on a HiSeq2500 (Illumina) using HiSeq Rapid SBS Kit v2 200 cycles (Illumina—FC-402–4021), HiSeq PE Rapid Cluster Kit v2 (Illumina—PE-402-4002), and HiSeq Rapid Duo cBot Sample Loading Kit (Illumina—CT-403–2001) following the manufacturer instructions.

#### Proximity ligation technology (hi-C sequencing)

To create both a reference *Daphnia* genome and a gut metagenome, we used the Hi-C technology (39). Two Hi-C libraries were generated using the Phase Genomics ProxiMeta kit version 4.0. Approximately 500 mg of *D. magna* (LRV0_1) tissue was crosslinked for 15 min at room temperature with end-over-end mixing in 1 ml of Proximo crosslinking solution. The crosslinking reaction was terminated with quenching for 20 min at room temperature again with end-over-end mixing. Quenched tissue was rinsed once with 1× Chromatin Rinse Buffer (CRB), resuspended in 700 μl ProxiMeta Lysis Buffer 1, combined with 500 ul glass beads and vortexed for 20 min. A low-speed spin was used to clear the large debris and the chromatin containing supernatant transferred to a new tube. Following a second higher speed spin, the supernatant was removed and the pellet containing the nuclear fraction of the lysate was washed with 1× CRB. After removing 1× CRB wash, the pellet was resuspended in 100 μl ProxiMeta Lysis Buffer 2 and incubated at 65°C for 15 min. Chromatin was bound to Recovery Beads for 10 min at room temperature, placed on a magnetic stand, and washed with 200 μl of 1× CRB. Chromatin bound on beads was resuspended in 150 μl of ProxiMeta fragmentation buffer and 11 μl of ProxiMeta fragmentation enzyme added and incubated for 1 h at 37°C. The beads were washed with 1× CRB and resuspended in 100 μl of ProxiMeta Ligation Buffer supplemented with 5 μl of Proximity ligation enzyme. Proximity ligation reaction was incubated at room temperature for 4 h with end-over-end mixing. To this volume, 5 μl of Reverse Crosslinks enzyme was added and the reaction incubated at 65°C for 1 h. After reversing the crosslinks, the free DNA was purified with Recovery Beads and Hi-C junctions were bound to streptavidin beads and washed to remove unbound DNA. Washed beads were used to prepare paired-end deep sequencing libraries using the ProxiMeta Library preparation reagents. Sequencing was performed on an Illumina HiSeq 4000, generating a total of 235051495 PE read pairs. Shotgun reads obtained from Hi-C libraries were filtered and trimmed for quality using fastp (40), normalized using bbtools (https://jgi.doe.gov/data-and-tools/software-tools/bbtools/) and assembled with MEGAHIT (41) using default parameters. The Hi-C data were analysed using the ProxiMeta platform (http://phasegenomics.com/technology/bioinformatics/).

#### Hybrid genome assembly

The short and long reads data obtained from different platforms were used for the *de novo* hybrid assembly of the *D. magna* genome (Supplementary Figure S1). The long reads obtained with ONT were used as a backbone to assemble scaffolds. The Illumina short reads were used to correct the ONT reads, which typically have higher error rate. The corrected long reads were mapped onto the Hi-C scaffold to obtain a chromosomal level assembly of the genome with high base call quality. The ONT reads were pre-processed using WTDBG2 and corrected with corErrorRate = 0.30 (42). The corrected long reads were assembled using the wtdbg2 assembler (43), mapped using Minimap2 v2.17 (44), and polished using Racon v1.4.3 (45). The Illumina short reads were introduced at this stage to improve the quality of the assembly. Before their use to polish the ONT reads, the Illumina-generated reads were demultiplexed using the bcl2fastq conversion software (Illumina), and pre-processed with Trimmomatic v0.39 (46), using the following options: ‘trimmomatic-0.39.jar PE -phred33 ILLUMINACLIP:adapter_seqs.fa:2:30:10 LEADING:30 TRAILING:30 MINLEN:50 HEADCROP:6″. QC plots were generated using fastqc (https://www.bioinformatics.babraham.ac.uk/projects/fastqc/). Jellyfish v2.2.6 was used to estimate K-mer counts (47) and Quake was applied for sequencing error correction (48). GenomeScope v2.0 was used to estimate genome size, repeat content and heterozygosity by K-mer based statistical approach (49). The cleaned and trimmed Illumina short reads were mapped onto the long ONT reads with Minimap2 (44). This step was followed by another polishing round with Racon v1.4.3 (45). Finally, Pilon v1.23 was used to identify and remove mis-assemblies, incorrectly called base pairs and gaps (50). The consensus genome from ONT and Illumina reads was converted into a fasta format for mapping onto the Hi-C reads. The Proximo algorithm was used to assign assembled scaffolds to super scaffolds, arrange contigs into a linear ordering, and orient contigs. This scaffolding algorithm was combined with a scaffold optimization process that performs tens to hundreds of thousands scaffolding attempts to find the super scaffold solution most concordant with the data. The Juicebox visualisation tool (https://github.com/aidenlab/Juicebox) was used to analyse and visualise the Hi-C mapped data.

The mitochondrial genome sequences were extracted from the long-read ONT assembly and mapped onto the most recent *D. magna* mitochondrial genome (51), using nucleotide blast with p-value cut-off <10^−5^ to assemble the full mitochondrial genome for the LRV0_1 isolate.

### Transcriptome data

Two sets of RNA sequencing (RNA-Seq) data were used to annotate the *D. magna* genes, as explained below. The annotated genes were mapped onto the genome and cross referenced with the previously published reference gene set for *D. magna* (22,52). The two RNA-Seq datasets were obtained from exposures to 12 environmental conditions and developmental stages obtained from nine morphological landmarks.

#### RNA-Seq data from 12 environmental exposures

The RNA-Seq data obtained from environmental exposures are part of a large experimental effort in which the impact of warming combined with food levels and carbamate insecticides was investigated on *D. magna* populations resurrected from Lake Ring, a lake with a well-known history of human impact over 100 years (30). The exposures mimicked environmentally relevant conditions that the lake experienced over a century and included temperatures of 18°C plus three (21°C) and six degree (24°C). These temperatures were projected increases according to the intergovernmental panel for climate change at the time of the publication (53). The two nutrient levels used (2.4 mgC/l and 0.2 mgC/l) mimicked eutrophic and oligotrophic conditions, respectively; the two concentrations of the insecticide Carbaryl (Pestanatal) (10 μg/l and 4 μg/l) were within the range reported in natural lakes to have a sublethal effect on *Daphnia* (54), with carbamate insecticides being among the topmost found in the lake (30). Combinations of the three temperatures with either nutrient levels or insecticide concentrations were also explored to assess the combined effect of multiple stressors on *Daphnia* fitness (31). RNA-Seq data were obtained from last instar whole animal tissue collected following exposure to the conditions explained above in two technical replicates. Total RNA was extracted with the RNA Advance Tissue kit (Beckman Coulter) and prepared into cDNA libraries with the NEBNext Ultra Directional RNA Library Prep Kit (New England Biolab E7420L), using the NEBnext Multiplex Oligos for Illumina Dual Index Primers (New England Biolabs E7600S), following the manufacturer guidelines. The libraries were sequenced on a HiSeq4000 by EnviSion (https://www.envision-service.com/) aiming to obtain 5 million reads per sample. Sequenced reads quality was assessed using fastqc (v0.11.5) (https://www.bioinformatics.babraham.ac.uk/projects/fastqc/) followed by multiqc (v1.5) (55). The RNA-Seq reads were mapped onto the *D. magna* reference transcriptome (22,52) and residual contaminant sequences, which may consist of residual gut bacteria and algae used as feedstock, were removed following blast searches in the NCBI database. The reads were then trimmed using Trimmomatic 0.36 (46) with the following parameters: (i) Illumina adapter cutoff with two seed mismatches; (ii) palindrome clip threshold of 30 and a simple clip threshold of 10; (iii) Phred quality score >30; (iv) minimum trimmed reads length of 50 bp. RNA-Seq reads were assembled into transcripts using Trinity assembler. Tblastn (56) and Exonerate (57) were used with the uniprot reference database (Uniref100) to identify protein coding sequences from these assembled transcripts.

#### RNA-Seq data from developmental data

The developmental RNA-Seq data were obtained from nine morphological landmarks (Supplementary Table S1) of genetically identical female and male clones of the *D. magna* isolate IRCHA 5 (Reading, UK). Male formation was induced by exposing parthenogenetic females to 100 nM of methyl-farnesoate (Sigma-Aldrich) dissolved in Dimethyl Sulfoxide (DMSO). Mature females were monitored hourly. The time of egg release into the brood pouch was considered time zero of the developmental landmarks. The second brood was used for the staging of the developmental landmarks. At each landmark, adult females were preserved in RNAlater, incubated at 4°C for less than 36 h and transferred to −80°C. Total RNA was extracted from the broods dissected from these adult females preserved in RNAlater using dissection needles under a laminar flow hood. All stages were washed with RNAse free water to remove residual maternal tissue. RNA extractions were performed using the Norgen Column RNA extraction kits on pooled embryos from single broods following a tissue lysing stage with ceramic beads. Stranded mRNA libraries were constructed from the extracted RNA using an Illumina TruSeq Stranded mRNA HT kit on at least 3 replicates per sex and stage. The Illumina PE 100 bp libraries were sequenced on an Illumina HiSeq 2500 platform aiming for 8 million reads per sample and replica.

### *Daphnia magna* gene set

*D. magna* genes were predicted from the isolate LRV0_1 using multiple independent evidence following the steps described below (Supplementary Figure S2):

Step 1 – Gene identification

We applied the Funannotate gene prediction and annotation pipeline (https://doi.org/10.5281/zenodo.4054262) to the hybrid *D. magna* assembly for gene identification (Supplementary Figure S3). The reliability of these gene calls was improved by using our own paired-end stranded RNA-Seq data obtained from the same genotype used for the genome assembly across 12 environmental conditions (see above). Prior to running the Funannotate pipeline, we used RepeatModeler2 (58) to *de-novo* identify transposable elements. We applied RepeatMasker (59) to mask repeat elements and low complexity DNA sequences, such as Long Terminal Repeats (LTR) using curated libraries of repeat motifs from Repbase (Genetic Information Research Institute; https://www.girinst.org/) and Dfam (60). Following the identification of transposable elements and the masking of low complexity DNA elements, we identified genes in the *D. magna* genome using multiple supporting evidence. These are:

- RNA-Seq data generated from the LRV0_1 isolate exposure to a suite of 12 environmental conditions described above were mapped to the masked hybrid genome assembly using TopHat to identify the splicing junctions between the exons. Repeat masked genome sequences and splice junctions obtained from TopHat were used for *ab initio* gene predictions using GeneMark-ET (61), Augustus (62) and SNAP (63).
- tRNAscan-SE 2.0 was used to predict transfer RNA (tRNAs), including training models to predict pseudogenes and mitochondrial tRNAs (64).
- Transcript models from GMAP (65) and PASA (66) were used to determine the gene structures and generate transcript models.

Step 2 – Independent gene validation

The identified genes from Step 1 were validated with independent evidence from either one or more (up to 4) gene modellers, orthology to genes from other species, orthology to genes from other *Daphnia* genes, and homology to protein domains in the Pfam database (Supplementary Figure S2).

#### Gene modellers

The genes identified by the Funannotate pipeline in Step 1 were validated and supplemented by four independent gene modellers: CLASS v2.1.7 (67), Scripture VPaperR3 (68) Stringtie v2.1.4 (69)) and geneid (70)); https://github.com/guigolab/geneidx). These modellers were trained on the developmental RNA-seq data obtained from nine developmental stages of *D. magna* female and male clones from the IRCHA 5 (Reading, UK) commercial isolate described above (Supplementary Table S1).

#### Orthology to genes in other species

Genes identified by the Funannotate pipeline in *Step 1* were also validated based on their orthology to genes found in other 50 taxa in OrthoFinder (71), Orthologer (72) and Broccoli (73) using default parameters. The taxa used for the orthology search are listed in Supplementary Table S2.

#### Daphnia orthology

The protein coding genes identified in Step 1 that are specific to the *Daphnia* genus were validated by searching for orthologs among the proteomes of four other *Daphnia* species (*D. pulex, D. pulicaria, D. obstusa* and *D. galeata* (37,74–77)*)* using blastp (78) and by applying a minimum identity of 60% and an e-value smaller than 1 × 10^−5^.

#### Protein domains

The genes identified by the Funannotate pipeline in *Step 1* were validated by the identification of protein coding genes using InterProScan (79) and eggNOG-mapper (80), which include PFAM, SwissProt, KEGG and GO databases.

#### Paralogs

Duplicated protein coding genes that are specific to the *Daphnia* lineage or that have diverged to the point of being overlooked in orthology searches were also considered here as supporting evidence. To identify the paralogs, blastp was used (78), applying a minimum identity of 60% and an e-value smaller than 1 × 10^−5^, against the proteome of the LRV0_1 *D. magna* isolate.

The mitochondrial genome annotation was performed using the most recent mitochondrial genome assembly as reference and employing blastn to align the sequences with p-values cutoff set at 1 × 10^−5^ (NIES (51)).

Step 3 – Consolidating gene prediction resources

The overall predicted genes by the Funannotate pipeline and the genes supported by independent evidence from Step 2 were mapped against the reference gene set of *D. magna* (22,52) based on *de novo* transcriptome assembly. Genes annotated in the LRV0_1 isolate were identical to the reference if they showed nucleotide identity larger than 70%, coverage larger than 70% and e-value smaller than 1 × 10^−5^. These genes are included in the submitted resources to NCBI including their location on the newly assembled *D. magna* genome with start and end position of each gene on the assembly. Genes predicted from the developmental RNA-Seq data not matching the criteria of identity and coverage but supported by at least 3 modellers (Supplementary Figure S4) were included in the *D. magna* gene set for the LRV0_1 isolate.

Step 4 – Gene annotation completeness

The completeness of the annotated *D. magna* gene set was assessed with BUSCO (81) using as reference the library of single copy ortholog genes from arthropods (v.odb9). A total of 1066 orthologous genes were used for this analysis. The protein coding genes predicted for the *D. magna* LRV0_1 isolate were compared to the one of previously published genomes, including the NIES (82) and the Xinb3 isolate (LRGB00000000). The KIT isolate (83) was not included in this analysis because gene functional annotations for this isolate were not available in public databases. In addition, the unique KO terms and the number of KEGG pathways across the NIES, Xinb3 and LRV0_1 isolates were compared. The protein-coding genes were annotated with KEGG ortholog terms (KO terms; (84)) and retained only if the KO terms were assigned consistently by BlastKOALA (85) and KofamKOALA (86). The assignment of KO terms to KEGG pathways was based on the *D. magna* NIES genotype (dmk; T07514), which is the current reference in the KEGG database.

### Epigenomic landscape

#### Methylation data

Whole genome bisulfite sequencing data were generated from 20 last instar clonal copies of the LRV0_1 genotype maintained under standard laboratory conditions (16:8 h light:dark regime, 20°C and 0.8 mg carbon/l of *Chlorella vulgaris* daily). The Epicenter MasterPure™ Complete DNA and RNA Purification Kit was used to extract genomic DNA. DNA quantity was assessed using a Qubit 3.0 Fluorometer (Invitrogen) and quality was visualised using a 1% agarose gel. Samples were sent to BGI Tech Solution Co., Ltd (Hong Kong) for bisulfite library preparation and sequencing. Directional bisulfite libraries were prepared by fragmenting genomic DNA to 100–300 bp by sonication. DNA-end repair was then carried out along with ligation of methylated sequencing adaptors. The ZYMO EZ DNA Methylation-Gold kit was used for bisulfite treatment with subsequent desaltation, size selection, PCR amplification and final size selection before sequencing. A 1% unmethylated lambda spike was included in the sequencing run to assess bisulfite conversion rates in downstream analyses. Sequencing was carried out on an Illumina HiSeq 4000 using 150 bp paired end reads, aiming for a depth of sequencing of 10X. Data quality was checked with Fastqc v.0.11.8 (https://www.bioinformatics.babraham.ac.uk/projects/fastqc/) and aligned to the hybrid reference genome described above using Bismark v.0.22.3 (87) with the ‘*filter_non_conversion’* mode. Reads were also aligned to the lambda reference (NCBI Accession: PRJNA485481) to determine the bisulfite conversion efficiency. Genome-wide DNA methylation levels were calculated as the total percentage of reads supporting a cytosine call in a CpG context minus the lambda conversion efficiency. Weighted methylation levels of genomic features were calculated as in Schultz *et al.* (88). Briefly, the weighted methylation levels were calculated as the number of CpGs in a given region (e.g. an exon) × the methylated reads in that region, divided by the number of CpGs × the total number of methylated and unmethylated reads. This is a more conservative estimate of methylation levels as compared to using only the percentage of cytosine reads within a region as it accounts for uneven coverage across positions. Individual CpGs, with a minimum coverage of 10×, were identified as methylated following a binomial test using the lambda conversion rate as the probability of success and correcting p-values using the Benjamini-Hochberg method (89).

A reciprocal protein blast was carried out to identify the genes involved in DNA methylation maintenance (DNMT1) and establishment (DNMT3a) using 321 insect DNMT1 gene sequences and 110 insect DNMT3a gene sequences from http://v2.insect-genome.com/Pcg, using blastp v2.2.3 (90). CpG methylation bias across all individual codons was assessed within the genome and each site was annotated with their codon degeneracy level, following (91), i.e. putatively neutral sites show four-fold-degeneracy (where any base polymorphism results in the same amino acid) and sites open to selection show zero-fold-degeneracy (where any base polymorphism results in a different amino acid). The relationship between DNA methylation and gene expression was then determined using custom R scripts as in (92) and the relationship between DNA methylation and open chromatin was determined using DeepTools v.3.1.3 (93). Gene ontology enrichment of highly methylated genes (>0.7 weighted methylation level, *n* = 170) and genes with no methylation (n = 3611) was carried out using the R package GOStats v2.56.0 (94). For highly methylated genes, all methylated genes (with a weighted methylation level higher than the lambda conversion rate, *n* = 7922) were used as a background set and for genes with zero methylation, all genes present in the methylation data were used as a background set (*n* = 18817).

#### ATAC open chromatin data

ATAC-Seq data were generated on the same tissue and exposures used for the transcriptome data above and following the modified protocol for ATAC-Seq for non-model species described in (95). Following DNA purification, libraries were prepared following (95). The quality and size of the ATAC libraries was assessed using a High Sensitivity D1000 Screentape. The libraries were quantitated on a Qubit 3.0 Fluorometer (Invitrogen) and sized using the Tapestation to determine the final molar concentration for sequencing. Illumina compatible paired-end DNA libraries were sequenced using Nextera primers and sequenced on an Illumina 2500 platform. The ATAC seq data were preprocessed following (95). Briefly, the Illumina sequence adapters and low quality reads were removed using Trimmomatic (v0.32) (46) using the following parameters: ‘ILLUMINACLIP:List_of_Adapaters.fa:2:30:10 LEADING:30 TRAILING:30 MINLEN:50 HEADCROP:10″. The reads that passed the quality threshold were indexed on the reference genome of *D. magna* described above, using Bowtie2 (v2.2.6) (96,97) ultra-fast aligner with the following settings: ‘-D 20 -R 3 -N 0 -L 20 -i S,1,0.50’. PCR duplicates and mitochondrial DNA were removed using the Broad Institute Picard tools (2.10.5) (http://broadinstitute.github.io/picard/) before calling ATAC peaks. MACS2 callpeak (98) was used to call the peaks from each sample and technical replicate; QC matrices were calculated on unmerged technical replicates. For comparative peak analysis replicates were merged.

### Metagenome assembly and functional annotation

Sequences obtained using the proximity ligation technology were masked with eukaryotic DNA from the LRV0_1 isolate to obtain a reference metagenome for *D. magna*. The prokaryotic genomes binning was performed using four binning algorithms (Proximeta (99), MaxBin2 version 2.2.7; (100); Metabat2 version 2.12.1 (101); and BinSanity version 0.5.4 (102)) (Supplementary Figure S5). The assembled genome fragments were dereplicated, aggregated and scored using DAS Tool version 1.1.4 (103). The completeness and contamination of the draft genomes were evaluated via CheckM version 1.1.3 (104) (Supplementary Figure S5). The completeness of the draft genomes was estimated by the occurrence and composition of singletons (single-copy marker genes), while the purity of the draft genomes was estimated by the frequency of singletons, following (104). The prokaryotic protein coding sequences (Prodigal, version 2.6.3), ribosomal RNAs (Barrnap, version 0.9), and transfer RNAs (Aragorn, version 1.2.41) of the assembled draft metagenome were annotated using Prokka (version 1.13) {Seemann, 2014 #109}.

All the sequences forming the metagenomes were mapped onto currently available *D. magna* genomes (GCA_020631705.2, GCA_001632505.1, GCA_003990815.1, GCA_927798085.1, and GCA_927798105.1) and *Chlorella vulgaris* assemblies (GCA_009720215.1, GCA_009720205.1, GCA_008119945.1, GCA_001021125.1, GCA_023343905.1) available in the NCBI database, to remove residual contaminant reads originating from the host and the feedstock (algae). Following this step, the prokaryotic protein coding sequences (CDSs) were predicted for the gut metagenome using Prodigal (version 2.6.3; {Hyatt, 2010 #110}). These CDSs were then aligned against the NCBI protein database (NCBI-nr) using the Diamond blastp algorithm (105), with the following criteria: >80% sequence similarity, query coverage >70%, and *E*-value <10^−5^. The CDSs were taxonomically assigned using the naïve LCA (lowest common ancestor) approach (106) based on the GTDB (Genome Taxonomy Database) taxonomy (107,108). They were functionally annotated using the SEED databases (109) in MEGAN6, set on long reads mode (version 6.23.2; (107)). The CDSs were aligned against publicly available databases to mine for antibiotic resistant genes [CARD (110)), ARDB (111), NDARO (112)] and virulence factors using [VFDB (113), Victors (114), and Patric VF (115)], using Diamond blastp and the same criteria used above for the protein coding sequence predictions. These reference genes databases were downloaded from PATRIC (version 3.6.12) (115).

## RESULTS

### The *daphnia magna* genome assembly and its genetic landscape

**Figure 1.**
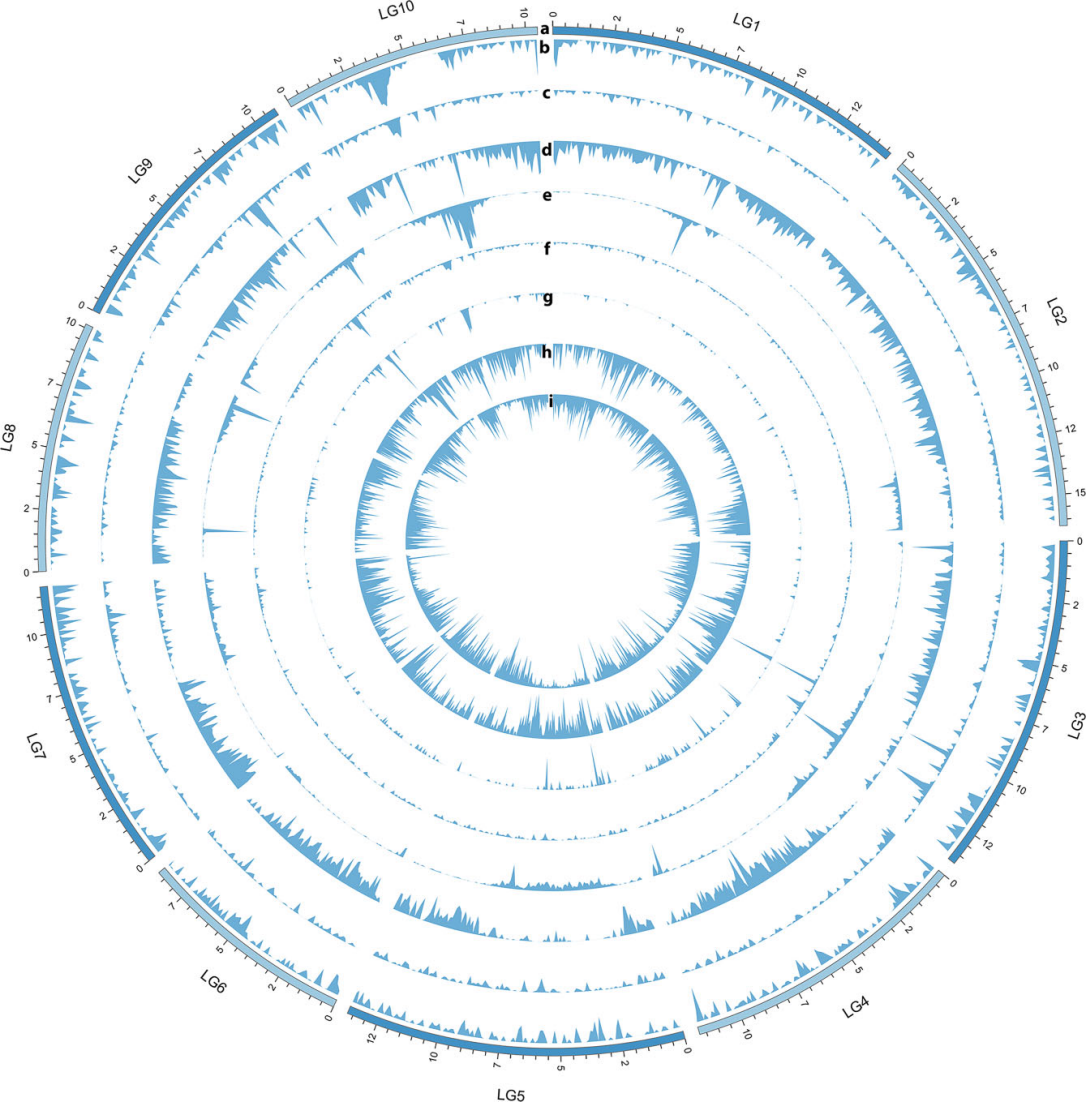
Circos plot. Overview of the *D. magna* genome, including (**a**) the 10 chromosomes identified with the Hi-C technology and supported by long reads Oxford Nanopore and short read Illumina reads (LG1–LG10 on an Mb scale); (**b**) GC content (%; range: 0–0.664); (**c**) gene density count (range: 0–0.088); (**d**) methylation level (% range: 0–0.062); (**e**) distribution of long terminal repeat (proportion of LTR; range: 0–0.594); (**f**) distribution of Long Interspersed Nuclear Elements (proportion of LINEs; range: 0–0.088); (**g**) distribution of Short Interspersed Nuclear Elements (proportion of SINEs; range: 0–0.133); (**h**) distribution of DNA transposons (%; range: 0–0.023); (**i**) distribution of simple repeats (proportion of SR: 0–0.125). A window size of 0.1 Mb was used for all genome properties plotted.

The chromosome-level *D. magna* genome assembly was obtained by combining Oxford Nanopore long reads (10551693 reads with insert length spanning between 7 and 48 kb), short Illumina reads (106023094; 250 bp paired-end reads) (Supplementary Table S3) and proximity ligation Hi-C data (Supplementary Figure S6). Genome characteristics identified with GenomeScope (116) using short read Ilumina data predicted a haploid genome length between 133054791 bp and 133206470 bp with repeat length between 36456744 and 36498304 bp (Supplementary Table S4; Supplementary Figure S7). The proximity ligation data confirmed the length of the genome and identified 10 chromosomes, which were mapped onto the most recent *D. magna* linkage map (117) (Figure 1a; Supplementary Table S5). The hybrid chromosome level assembly was composed of 71 scaffolds for a total genome length of 130 Mb (BUSCO genome completeness of 99%; Table 1). The complete and single copy genes found in the ortholog database comprised 96% of the total genes; 3% of the genes were complete and duplicated. Only 0.7% was found to be fragmented and 0.6% missing. The N50 of the hybrid genome assembly was 12 Mb; the GC content was 40% (Figure 1b), comparable to the one found in previous assemblies (Table 1). Gene density was evenly distributed across the genome with occasional peaks in LG9, LG10 and LG3 (Figure 1c). The repeat content was 40.37 Mb (31.07% of the genome) with the majority (28.48%) of these repeats being interspersed - dispersed throughout the genome and nonadjacent (Figure 1e–i). DNA transposons (Figure 1h) comprised 13.01% (Figure 1i) and retroelements comprised 2.24% of the genome (Figure 1h; Supplementary Table S5). Almost 13% of the transposable elements (TE) were unclassified (Supplementary Table S6). The assembled mitochondrial genome comprises 14513 bp and consists of 13 protein coding genes, 22 tRNAs, 2 rRNAs and 33% GC content.

**Table 1.** Assembly metrics and annotation statistics. Metrics for the *D. magna* genome assembly and gene annotation from this study (LRV0_1) and previous genomics studies (*D. magna* 2.4v; KIT and NIES isolates). n/a: the *D. magna* 2.4 does not have a gene annotation. Values in brackets for the LRV0_1 isolate refer to the reduced gene set supported by multiple lines of evidence

	LRV0_1 (this study)	Xinb3-*D. magna* 2.4 (NCBI: LRGB00000000)	KIT (Lee *et al.* 2019)	NIES (Byeon *et al.* 2022)
No scaffolds	71	3404	4193	493
Largest chromosome/scaffold (bp)	16 263 983	3 718 170	16 359 456	19 099 978
Length of 10 chromosomes (bp)	122 644 335	n/a	122 940 627	173 470 305
Total length (Mb)	129.9	131.2	113	173.5
N50 (Mp)	11.9	0.5457	10.1	12.5
GC content (%)	40.18	40.59	40.54	39.81
Gene number	31 337 (26823)	26 646	15 721	16 436
Average gene length (bp)	1811 (2148)	n/a	2407	3091
Average CDS length (bp)	1008 (1113)	1273	1394	1720
BUSCO (%)	99	96.2	96.7	95.4

**Figure 2.**
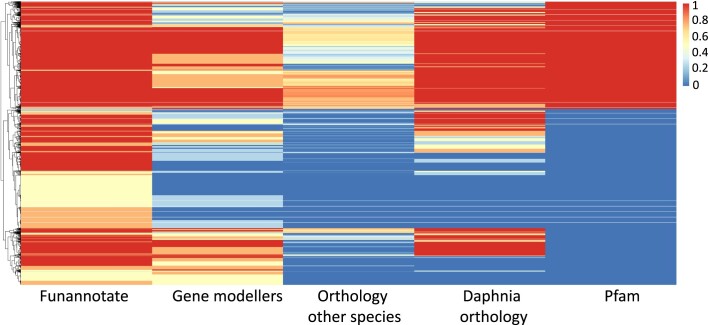
*Daphnia magna* gene set. Independent pieces of evidence supporting the gene identification in the LRV0_1 isolate. ***Funannotate—***genes identified by the Funannotate pipeline, using PASA, GeneMark, SNAP and Augustus. These data were trained on RNAseq obtained from 12 environmental exposures. The colour gradient shows genes supported by four modellers (red) to genes supported by none of the modellers (blue). ***Gene models—***genes validated by one or more modellers from CLASS, geneid, Scripture, and Stringtie. These data are trained on an independent set of developmental RNAseq data based on 9 morphological landmarks. The colour gradient shows genes supported by one (blue) up to four gene modellers (red). ***Orthology to other species—***genes supported by orthology to 50 other animal and plant species ranging from species supported by one (blue) up to 50 species (red). ***Daphnia orthology—***genes supported by orthology to other *Daphnia* species (*D. galeata*, *D. pulex*, *D. obtusa* and *D. pulicaria*). ***Pfam***- genes showing homology to protein domains in the Pfam database (1: red/0: blue). This figure was plotted using pheatmap v1.0.12 in R v4.0.2.

The Funannotate pipeline identified 33951 genes, of which 31337 were protein coding genes (CDS) and 2614 were tRNA genes. Of these protein coding genes, 24744 (79%) were supported by at least one and up to four gene modelers (Supplementary Figure S4; CLASS, scripture, Stringtie, and geneid); 16617 (34%) by orthology to genes in other species (Supplementary Table S2); 20474 (48%) by orthology to gene in other *Daphnia* species (Supplementary Table S2); and 11843 (38%) by protein family domains in the Pfam database (118) (Figure 2). A total of 5752 genes were predicted without recognisable protein domains, nor homology to other proteomes (Supplementary Figure S2; Supplementary Table S2). In total, 26823 genes were validated by these multiple lines of evidence (Supplementary Figure S2; Supplementary Table S2). Of these, 10975 genes were supported by five independent pieces of evidence; 4752 genes were supported by four pieces of evidence; 4423 genes were supported by three pieces of evidence; 6673 genes were supported by two pieces of evidence (Supplementary Figure S2). A total of 4506 genes were supported by a single piece of evidence. A total of 8 genes were predicted *ab initio* but rejected by the Funnannotate pipeline. An additional set of 7606 putative novel genes was identified by the gene modellers trained on developmental RNA sequencing data (Supplementary Table S7). We expected these genes to not likely have been identified in the previously published *D. magna* gene set or by the Funannotate pipeline run on the LRV0_1 isolate. This is because the latter two gene sets originate from adult or subadult whole animal tissue. Of the 7606 putative novel genes, 3689 genes were supported by four modellers (CLASS, scripture, Stringtie, and geneid); 3917 genes were supported by three modellers; 13305 genes were supported by two modellers; and 9059 were predicted by a single modeller (Supplementary Figure S4; Supplementary Table S7). Of the total set of putative novel genes, 894 were found to be also predicted by the Funannotate pipeline (blastn results; Supplementary Table S7), reducing the number of putative novel genes to 6712, 1223 of which were found to have high similarity to genes present in the previously published gene set (52). Following this filtering process, the number of novel genes originating from the developmental gene set was 5489 (Supplementary Table S7).

To assess the robustness of the genome assembly generated here, we quantified the overlap between genes identified by the Funannotate pipeline for LRV0_1, anchored on the assembly, and the previously published gene set for *D. magna*, based on *de novo* transcriptome assembly which comprises 29134 genes (52). The previously published gene set and the LRV0_1 gene set shared 20018 (64%) coding genes. If only genes supported by other independent pieces of evidence were considered (26823) the overlap between the LRV0_1 gene set and the previously published gene set was 71%. These genes map in a single unequivocal location on the genome assembly, whereas single genes in the LRV0_1 gene set may map to more than one predicted gene in the previously published gene set (Supplementary Table S8). This is likely due to the published gene set using alternative transcripts for gene annotation and to split genes. The genes shared between the LRV0_1 isolate and the previously published gene set were assigned to 5331 unique KO terms and 5310 total KO terms, amounting to 97% shared KO terms between the published reference gene set and the gene set generated for LRV0_1 (Supplementary Table S8).

To assess how the newly assembled genome compared to other published genomes of *D. magna* to date, we quantified KO terms and KEGG pathways in all sequenced genomes. The number of protein-coding genes in the LRV0_1 isolate was higher than both the NIES and the Xinb3 genotype, whereas the genes supported by multiple pieces of evidence in the LRV0_1 isolate were larger than the NIES isolate and lower than the Xinb3 isolate (Table 2). The number of genes with KO terms and unique KO terms in the LRV0_1 isolate were lower than both the Xinb3 and the NIES isolates (Table 2). The number of KO terms and unique KO terms did not differ between the overall 31337 protein coding genes and the reduced gene set supported by multiple pieces of evidence in the LRV0_1 isolate (Table 2). Notwithstanding the difference among the predicted KO and unique KO terms across the *D. magna* isolates, the number of KEGG pathways predicted was the same for all isolates (*N* = 130; Table 2).

**Table 2.** Functional annotation of *Daphnia magna* isolates. Functional annotation of the *Daphnia magna* strains NIES and Xinb3, compared to the LRV0_1 in this study, including the whole set of identified genes by the Funannotate pipeline and the reduced gene set supported by multiple lines of evidence. The total number of protein-coding genes, the total number of KO and unique KO terms, as well as the total number of KEGG pathways are listed

	NIES (Byeon *et al.* 2022)	Xinb3 (Orsini *et al.* 2016, 2018)	LRV0_1 (Funannotate; this study)	LRV0_1 (reduced gene set; this study)
Number of protein-coding genes	16 891	29 127	31 337	26 823
Number of genes with KO term	7966	9836	7551	7551
Number of unique KO terms	5569	5627	5382	5382
Number of KEGG pathways	130			

The newly assembled *D. magna* reference genome and the associated gene set are available in NCBI (NCBI BioProject ID: PRJNA727483 and PRJNA777104; the genome accession number is JAOYFB000000000). The genome and reduced gene set have also been submitted to the Alliance of Genome Resources (https://www.alliancegenome.org/), a consortium of seven model organism databases and wFleaBase (http://wfleabase.org/), hosting *Daphnia* genomics resources. All these resources were created to make the reference genome and associated gene set searchable and accessible to the wide research community.

### Epigenomic landscape

**Figure 3.**
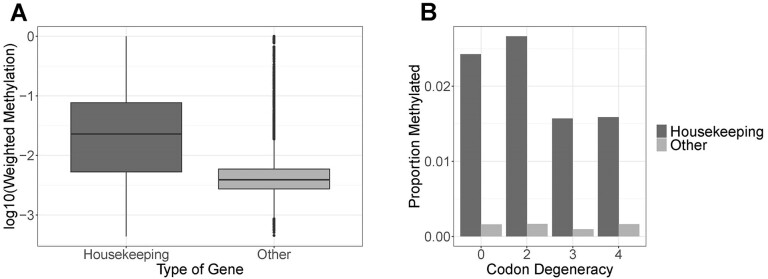
DNA methylation. (**A**) Boxplot showing the weighted methylation levels of putative housekeeping genes (*n* = 7225) compared to other confidently annotated genes (other), of which DNA methylation data are available (n = 11592). The black dots represent outliers beyond 1.5× of the interquartile range (*t*-test: *t* = 35.614, df = 8120, *P* < 0.001). (**B**) Barplot showing the proportion of methylated sites per codon degeneracy level for all codons within a gene, separated into putative housekeeping genes and all other genes (other). Within housekeeping genes codon degeneracy levels show significantly different propensities for being methylated (logistic regression: X_2_= 303.2, df = 3, *P* < 0.001).

We characterised the epigenomic landscape of *D. magna* by studying DNA methylation, open chromatin and gene expression patterns in the same isolate used for the genome assembly and gene annotation (final 12× coverage). The genome-wide DNA methylation level was 0.4% in a CpG context, with 0% found in CHG and CHH contexts (Figure 1d), comparable to previous reports in this species (119–121). Although we did not use replicates in our study, the level of methylation observed in the LRV0_1 isolate was comparable to the one of other 39 isolates of *D. magna* (unpublished data). The isolates will feature in an upcoming population-level methylation analysis. This level of DNA methylation is typically enriched in highly expressed genes with stable gene expression levels, thought to be housekeeping genes (122). Given the extensive RNA-Seq data sets used in this study from nine morphological landmarks of a female and a male clone (Supplementary Table S1) and environmental exposures described in methods, we were able to identify putative housekeeping genes - genes expressed in all conditions and datasets (n = 7225) - and quantify differential methylation levels between these and all other annotated genes with detectable gene expression (*n* = 11592). The putative housekeeping genes showed higher levels of DNA methylation than all other genes (Figure 3A, *t*-test: *t* = 35.614, df = 8120, *P* < 0.001).

We studied the relationship between DNA methylation and codon degeneracy across the genome. In addition, we examined this relationship separately for housekeeping genes and all other annotated genes. Codon degeneracy had a significant effect on methylation status (X_2_ = 303.2, df = 3, *P* < 0.001, Figure 3B), with an interaction between codon degeneracy and putative house-keeping status (X_2_ = 25.5, df = 3, *P* < 0.001), meaning that DNA methylation was higher in putative housekeeping genes. We then performed pairwise comparisons between codons with different levels of degeneracy using a Tukey's HSD test, which corrects for multiple testing. This analysis showed that in putative housekeeping genes zero-fold degenerate sites were more likely to be methylated compared to three- (*z* = 4.423, *P* < 0.001) and 4-fold sites (*z* = 15.397, *P* < 0.001) and less likely to be methylated than 2-fold sites (*z* = –3.368, *P* = 0.0173). Additionally, 2-fold sites were more likely to be methylated than three- (*z* = 5.262, *P* < 0.001) and four-fold sites (*z* = 15.045, *P* < 0.001). These differences were not observed in non-housekeeping genes (Supplementary Table S9).

DNA methylation was differentially enriched among coding regions (Supplementary Figure S8A, Kruskal–Wallis: X_2_ = 9910.1, df = 6, *P* < 0.01), with exons two to four having the highest methylation as compared to exons one, five and six (Supplementary Figure S8A, B; Supplementary Table S9). Additionally, exons showed considerably higher levels of DNA methylation compared to background intergenic levels (Supplementary Figure S9). DNA methylation was depleted in transposable elements as compared to background intergenic methylation levels (Supplementary Figure S9, Supplementary Table S10), Dunn post hoc with Benjamini-Hochberg correction: intergenic—TE: *Z* = –2.74, *P* < 0.01). Using a reciprocal blast search against a large database of insect DNA methyltransferases (DNMTs) we identified three isoforms of DNMT1 (Dmagna004434-T1, Dmagna004434-T2, Dmagna004434-T3), involved in DNA methylation maintenance, and two orthologs of DNMT3a (Dmagna014617-T1 and Dmagna0144665-T1), involved in *de novo* DNA methylation. In line with previous research (123), we find that the first ortholog lacks the pwwp domain, whereas the second has the domain. Nguyen *et al.* (123) suggest the pwwp-lacking ortholog is still catalytically active as it resembles the active DNMT3C found in mammals. A knockdown of these genes would be beneficial to determine the importance of their role in *Daphnia* DNA methylation. Finally, a gene ontology enrichment analysis revealed that both highly methylated genes (>0.7 weighted methylation level) and genes with no methylation were involved in a variety of cellular and metabolic processes (Supplementary Table S11).

Previous research suggested that DNA methylation in arthropods is associated with open chromatin through the recruitment of histone acetylation (124). To test whether this association is also found in *D. magna*, we examined the relationship between genome-wide DNA methylation and open chromatin patterns in the LRV0_1 isolate. We found no clear relationship between DNA methylation levels and open chromatin (determined via ATAC-Seq) at a genome-wide scale (Supplementary Figure S8C). However, given the differential level of DNA methylation across gene-bodies in *D. magna* we examined this relationship further, looking only at open chromatin peaks which fall over a gene. This inspection revealed peaks of DNA methylation within open chromatin regions, in addition to small peaks of DNA methylation flanking these regions (Supplementary Figure S8D). This relationship is consistent with the idea of DNA methylation being specifically associated with gene expression, i.e. genes found in open chromatin regions.

### *Daphnia magna* metagenome and its functional role

As the Hi-C technology (39) was applied on the whole animal, we were able to reconstruct a draft gut metagenome, as well as the host genome, from the same proximity ligation data. We used 82317 Hi-C reads to reconstruct the *D. magna* metagenome, following the masking of sequences mapping onto the reference genome of the host (*D. magna*; 40258 reads) and the algal feed (*Chlorella vulgaris*; 2590 reads). From these reads we assembled eight prokaryotic genomes. The draft genomes were made up of two main phyla: *Proteobacteria* and *Bacteroidota* (Table 3). Three of the prokaryotic genomes with high completeness were assigned to the order *Burkholderiales* (phylum *Proteobacteria*, class *Gammaproteobacteria*), two were assigned to order *Cytophagales* (phylum *Bacteriodota*), one was assigned to the class *Deltaproteobacteria* (phylum *Proteobacteria*), and one was assigned to the phylum *Bacteriodota*. One draft genome showed high purity (99.91%) but no match to known prokaryotic genomes (Table 3). The completeness of the bacterial genomes ranged between 60.72% and 96.41%, and purity ranged between 86.76% and 99.91% (Table 3). The size of the eight draft prokaryotic genomes ranged between 0.74 to 5.94 Mb (Supplementary Table S12). Between one and three ribosomal RNAs were detected in these genomes, as expected (125) (Supplementary Table S12). The number of tRNA detected in individual draft genomes varied between 17 to 65, in line with the theoretical expected tRNA abundance in prokaryotic genomes (between 20 and 62) (126). The tmRNA, involved in both transfer and messenger RNA’s functions, ranged between 1 and 2 and was not detected in two genomes. However, this may be due to biases present in the Aragorn tmRNA database used for tmRNA annotation, which only includes 57 bacterial genomes (127) (Supplementary Table S12).

**Table 3.** *Daphnia magna* metagenome. List of metagenomes isolated from the gut microbiota of *D. magna*. The microbial taxa, the completeness (%) and the purity levels (%) of eight draft genomes binned using the DAS tool are shown. The purity of the assemblies is calculated with CheckM

Bacterial taxon	Completeness (%)	Purity (%)
Cytophagales (order)	96.41	98.94
Burkholderiales (order)	99.36	99.02
Burkholderiales (order)	96.24	86.76
Cytophagales (order)	98.70	96.60
Burkholderiales (order)	89.89	90.80
Deltaproteobacteria (class)	42.65	99.43
Bacteroidota (phylum)	92.45	97.78
Bacteria (kingdom)	60.72	99.91

**Figure 4.**
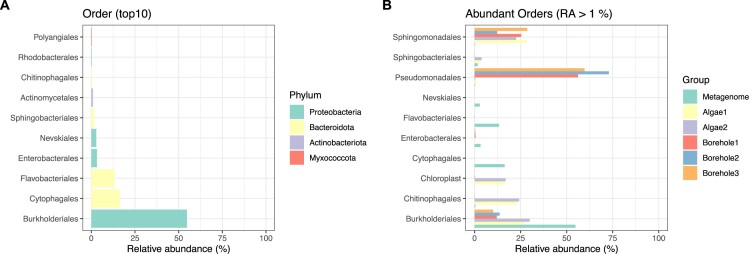
Taxonomic composition of the *Daphnia magna* metagenome. (**A**) Relative abundance (RA in %) of the 10 most abundant bacteria orders in the *D. magna* microbiome. (**B**) Order-level taxonomic composition of the *D. magna* metagenome, plotted against the algal feed in duplicates (Algae1 and 2), and the growth medium in triplicates (borehole). Only occurrences with relative abundances >1% were used in this plot. Plots in this Figure are supported by data in Supplementary Table S13.

**Figure 5.**
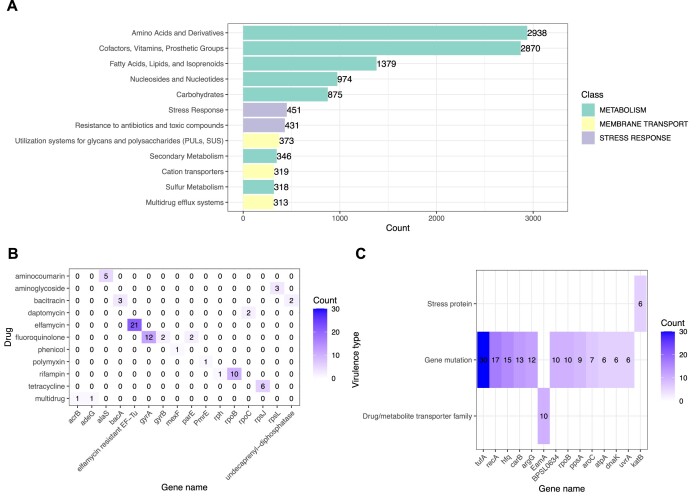
Functional annotation of the *Daphnia magna* metagenome. (**A**) Top 12 most abundant functional classes annotated in the *D. magna* metagenome; the colour coding of each bar is based on the functional class - metabolism (green), membrane transport (yellow), stress response (purple). (**B**) Number of identified antibiotic resistant genes (ARGs) plotted against the targeted antibiotic. **(C)** Numbers of detected virulence-related genes (virulence factor, VF) plotted against the virulence mechanistic class. The plots in this Figure are supported by data in Supplementary Table S14.

Within the *Proteobacteria* phylum, comprising 64.22% of the gut community, the order *Burkholderiales* made up 54.8% of the metagenome, whereas the phylum Bacteroidota made up 33.5%, and comprised two orders at relatively high abundance: *Cytophagales* (16.30%) and *Flavobacteriales* (13.30%) Figure 4A; Supplementary Table S13). These bacterial genomes were distinct from the ones of the growth medium (borehole water) and the algal feed (Figure 4B; Supplementary Table S13). The functional annotation of the metagenome was performed on 132015 putative protein coding sequences (putative CDSs); the numbers of CDS per genome ranged between 736 (unannotated genome) and 5514 (*Burkholderiales*) (Supplementary Table S12). The top 12 functions identified by the CDS include: amino acid biosynthesis, metabolism of vitamins, lipids, and nucleotides, carbohydrates and sulphur, membrane transporters for drug efflux (multidrug efflux systems), stress response genes, and resistance genes (e.g. antibiotic resistance) (Figure 5A). A total of 73 putative protein coding genes identified in the gut microbiota of *D. magna* were genes resistant to 10 common antibiotics (Figure 5B; Supplementary Table S14, AMR). Of these genes, 21 were elfamycin resistant, 14 were floroquinolone resistant and 11 were rifampin resistant (Figure 5B; Supplementary Table S14, AMR). A total of 286 CDSs were assigned to 90 genes, which could be assigned to 12 virulence or pathogenic factors (Figure 5C; Supplementary Table S14, VF). The top 14 genes among those were largely classified as gene mutants, followed by drug transporters and stress proteins (Figure 5C).

## DISCUSSION

### The improved genome assembly and annotated gene set of *daphnia magna*

Our aim to have a much-improved *D. magna* genome assembly proved successful. We obtained a chromosomal level assembly divided in only 71 scaffolds with an average length of 129.98 Mb. The genome length for *D. magna* was 130 Mb with a genome completeness of 99%, 40Mb smaller than the most recently published *D. magna* genome (NIES; (82)). This discrepancy was likely explained by the higher fragmentation of the NIES genome, leading to an overestimation of the genome size. The estimated genome size based on the K-mer and anchoring of the assembly on the Hi-C proximity data in our study makes the genome estimate robust. Our genome assembly is a significant improvement from previous assemblies, which were more fragmented with the number of scaffolds ranging between 493 (82)) and 40356 (LRGB00000000). The N50 of our hybrid genome assembly was 12Mb, comparable to the NIES assembly (82)) based on long read Oxford Nanopore technology, but significantly larger than previously assembled genomes (e.g. KIT (83)). The GC content in our assembly was comparable to the one found in previous assemblies.

The first assembled genome of the genus *Daphnia* was the *D. pulex* TCO genome (37). The estimated length of this genome was 200Mb with 31000 annotated genes. A new genome assembly for *D. pulex* was recently released based on a combination of paired-end and mate-pair Illumina sequencing libraries, providing better contiguity (77). The estimated genome size based on this assembly was 159 Mb supported by 96 scaffolds (77). Long-reads and improved contiguity have ameliorated the genome assemblies in invertebrates, including the well-established model species *Drosophila melanogaster* (128). The size of the *D. magna* genome presented here is comparable to the one of other *Daphnia* species, whose genome is published (*D. pulex*, 159 Mb (77); *D. galeata*, 133 Mb (76) and *D. pulicaria*, 156 Mb (74). Conversely, the number of annotated genes in these genomes fluctuates depending on the pipeline used and whether RNAseq or other independent evidence for gene functional annotations were used. *D. magna* (present study) and *D. pulex* (77), in which RNA-Seq was used as evidential support, have a comparable number of annotated genes. Conversely, when annotation only relies on gene modellers the total number of annotated genes sits around the 15000–17000 mark (74,76,82).

We identified a total of 31337 protein-coding genes, using the Funannotate pipeline, and an additional 5489 putative novel genes, supported by the developmental gene set. These putative novel genes are likely explained by early developmental genes, male-specific genes and the genotype used, which is distinct from LRV0_1. From the total genes identified by the Funannotate pipeline, a reduced set of 26825 genes was supported by other independent pieces of evidence, including gene modellers, RNA-Seq data from developmental data, orthology to genes in other species and protein family domains predictions. We annotated a higher number of genes than the recently published *D. magna* genomes, namely the KIT genome (83), for which 15721 gene were annotated, and the NIES genome (82), for which 16436 genes were annotated (of which 15684 functionally annotated). The number of annotated genes in our gene set is higher than the previously published gene sets for *D. magna* based on the *de novo* transcriptome by ca. 200 genes. The published gene set based on *de novo* transcriptome assembly (22,52) and the gene set derived from the LRV0_1 isolate share 71% of genes uniquely mapped onto the new genome assembly. The proportion of genes not shared between the gene sets may be explained by the genetic distance between the isolates and the gene annotation pipelines used. In both the NIES and the KIT gene annotation, the MAKER3 pipeline was used, resulting in 15000–16000 genes with known function. The higher accuracy of our approach can be appreciated from the number of genes common across *Daphnia* species. Only 7080 genes were found to be common to all *Daphnia* species in previous genome assemblies (82,83), whereas in our study the number of orthologs among all *Daphnia* species was 20474. Importantly, we are confident to have captured the functional landscape of *D. magna* by showing that the entire gene set predicted by the Funannotate pipeline and the reduced gene set supported by multiple lines of evidence have the same number of unique KO terms. The higher number of predicted KO terms from a smaller number of protein-coding genes in previously published *D. magna* genome annotations suggest a higher number of incomplete genes in previous annotations. This is confirmed by the equal number of KEGG pathways predicted across the published genomes and including the LRV0_1 isolate (N = 130 pathways). A caveat of our assembly is that a proportion of genes present in the previously published gene set was not found in the reference genome for the LRV0_1 isolate. This may be explained with genetic divergence between isolates or by incomplete assembly. Even if long reads are expected to provide better contiguity and assembly, it has been shown that GC rich proximal promoter regions, as well as highly repetitive genomic regions may be more challenging to assemble even in long-read based assemblies (129). With continuous developments in bioinformatic tools, we can expect further improvement of the *D. magna* assembly in the future. Regardless of future improvement of the assembly and reference gene set, the improved resources presented here will be useful to the community. This is particularly relevant given the broadening role of *D. magna* as model species to include applications as an early warning system and as a bioremediation agent for chemical pollution (21). The improved *D. magna* genome assembly and gene set presented here will empower the use of *Daphnia* as a diagnostic agent to identify adverse effects of chemicals for environmental and human health. The gene set developed using ecologically relevant biological perturbations is advantageous to connect toxic and physiological responses, whereas traditional toxicology approaches using concentrations of chemicals rarely encountered in the natural environment prevent the identification of early onset biomarkers of toxicity. The additional information provided by the gene set presented here, combined with already existing resources, will pave the way to comparative genomics analyses and synteny among *Daphnia* species for an in-depth understanding of chromosome-scale evolution, association studies, and quantitative trait loci mapping.

### DNA methylation in *D. magna* genome evolution

The function of gene-body DNA methylation is debated (130) with two main theories put forward by scientists. One theory suggests that DNA methylation acts as a stabilising factor, supported by enrichment of DNA methylation in housekeeping genes (131) (Supplementary Figure S10A). However, recent findings show that only about half of housekeeping genes annotated in arthropods are highly methylated (132). This pattern may be explained by the association of the DNA methylation with nucleosome occupancy. In arthropods, the presence of DNA methylation promotes the recruitment of H3K27ac (epigenetic modification to the DNA packaging protein histone H3) which, in turn, promotes gene expression through open chromatin (124).

Another theory suggests that DNA methylation is a mutagen itself and can provide genetic substrate for selection (Supplementary Figure S10B). This theory stems from the link between levels of DNA methylation and environmental change and the heritability of methylated sites in some species (133). It is also supported by the knowledge that DNA methylation deaminates cytosines to thymines at a 10-fold higher frequency than the genome-wide average (134), providing a substrate for increased mutation within these regions. Observed enriched DNA methylation at zero- and two-fold degenerate sites, and correlation between changes in gene-body DNA methylation and changes in gene expression are expected if DNA methylation acts as a mutagen. Whereas these patterns are observed in some species, they are not ubiquitous in nature and are rarely observed in arthropods (135).

We show a trend of increased DNA methylation in open chromatin peaks covering genes. We also find an enrichment of DNA methylation at zero-fold and two-fold degenerate sites. This evidence suggests that gene-body DNA methylation acts as a mutagen in *D. magna*, providing a substrate for selection through induced genetic changes at genome-wide scale. This mechanism is also parsimonious with DNA methylation being associated with broadly expressed genes and open chromatin regions. However, we also observe that DNA methylation is higher in housekeeping genes, which favours a stabilising role of gene-body methylation in *D. magna*. This pattern is found in other invertebrates, in which genes with housekeeping, constitutive and ubiquitous functions tend to be more methylated than those with inducible functions (136). There are two potential limitations to our study that may contribute to these inconclusive findings on the role of DNA methylation in genome evolution. First, the methylation patterns were studied in the whole animal, whereas methylation may differ across animal tissues. Secondly, to confirm the role of gene-body methylation as mutagen, potentially involved in adaptive processes, population-level whole genome-DNA methylation studies are needed. DNA methylation has been shown to play an important role in species adaptation and acclimation to environmental change through induced genetic changes (130). Specifically, differential DNA methylation patterns were correlated with genetically divergent populations adapted to different environmental conditions (130). Future population-level analysis of genetic and epigenetic patterns in *D. magna* will provide key insights into the highly debated role of methylation as a stabilising or mutagenic factor in genome evolution. By studying temporal populations that originate from the same genetic pool and respond to environmental change over time, important insights can be gained on the role of genetics and epigenetics in adaptation to environmental change, whereas controlled experimental transgenerational exposures can provide important insights into reversible epigenetic patterns leading to acclimation/plastic responses.

### The microbiota plays a key role in *D. magna* metabolic and defence functions

All eukaryotes spend their lives associated with communities of microorganisms, which play an important role in nutrition, development, metabolism, behaviour and disease resistance (137–139). To fully understand an organism's biology, it is vital to assess the role of the microbiota in modulating the biological functions of the host (140). The *D. magna* microbiome has been shown to be genotype-specific (23), responsive to environmental change (137,140–143) and involved in important fitness and metabolic functions (142,144). The use of proximity ligation technology enabled us to define the genomic content of *D. magna*-associated bacteria and their metabolic functions to support the host fitness. We identified eight draft bacterial genomes, one of which was not found in existing databases. The lack of records in published databases suggests that this genome may be novel. However, the draft genome was incomplete, made up of 89 contings. Therefore, a firm conclusion on its novelty could not be reached. A blast search of the 16S rRNA could have helped resolve the taxonomy of this draft genome. However, the 16S region was not found in the annotated contigs of this bacterial genome, highlighting its low level of completeness. Additional sequencing effort is required to fully resolve any novel genome in the gut microbiota of *D. magna*.

Different mechanisms of acquisition of the gut microbiota have been proposed for *D. magna*. Transplant experiments on adult isolates purged of their gut microbiota and exposed to a donor suggest that the *D. magna* microbiota is vertically transmitted and genetically determined (23). Conversely, assessment of the gut microbiota transplant through fitness responses rather than direct quantification of the gut community via the metagenome analysis suggests horizontal transmission of the microbiota (143). The top three abundant orders of bacteria found in our study to populate the *D. magna* gut community (*Burkholderiales*, *Cytophagales*, and *Flavobacteriales*) were not found in the growth medium nor in the algal feed. The orders *Burkholderiales and Flavobacteriales* were found in the algal feed and the guts. However, their relative abundance was significantly lower in the algal feed than in the *Daphnia* microbiota. Similarly, *Pseudomonadales* and *Sphingomonadales* were only found in the growth medium. These results support that the gut microbiota is vertically transmitted and genetically determined.

The gut microbiota community is intimately linked to the physiology and fitness of the host (145). In *Daphnia*, the gut microbiota has been previously shown to regulate metabolism, detoxification and population fitness (23,146,147). Here, we reveal the role of the gut microbiome in antibiotic resistance and virulence/pathogen response. Antibiotic resistance, driven by the overuse and misuse of antimicrobials (148), may arise from a variety of mechanisms, particularly horizontal gene transfer of virulence and antibiotic resistance genes, (149). For the first time, our study provides evidence of resistance to commonly used antibiotics in the *Daphnia* microbiome, which may facilitate *Daphnia’* adaptation to antibiotics in the environment. Of particular interest are the elfamycin, rifampin and fluoroquinolone resistance genes. By inhibiting EF-Tu activity, elfamycin can disrupt the process of decoding and translating mRNA (150). Rifampin is a derivative of the broad-spectrum antibiotic rifamycins and acts as an inhibitor of RNA synthesis (151). Fluoroquinolone-resistant gyrA genes have been shown to cause multidrug resistance in humans (152). In *Daphnia* fluoroquinolones can induce both acute and chronic adverse effects, even at concentrations below regulatory approved thresholds detected in rivers, lakes, and urban outlets (153). Also detected in the draft bacterial genome of *D. magna* is a tetracycline resistance gene; tetracycline has been shown to reduce fecundity in *D. magna* (154). These results suggest that defence mechanisms that require a fast pace of evolution are likely mediated by the evolution of the gut microbial community in *Daphnia*. This result is supported by the virulence factors identified in our study, including at least 14 gene mutants. Mutations induced by selection in these genes have been associated with reduced pathogenicity from chronic infections (155), multidrug resistance (e.g. via the drug/metabolite transporter (DMT) superfamily) (156), and enhanced tolerance to oxidative stress (e.g. through the katB gene) (157).

## CONCLUSION

In this study, we leverage the properties of *D. magna* to characterise the holobiome of this premier model species in ecology and ecotoxicology, elevating this species to the role of model in environmental genomics. The chromosomal-level assembly of the *D. magna* genome presented here, complemented by the assessment of the host and metagenome functional landscape, as well as the potential mutagenic role of DNA methylation, provide important insights into the mechanisms of genome evolution in a highly plastic organism capable of rapid genetic evolution. The resources presented here will be vital for comparative genomics and phylogenomics analyses, contributing to our understanding of genomic and epigenomic mediated evolution in response to rapidly changing environments.

## Supplementary Material

gkad685_Supplemental_Files

## Data Availability

The Illumina short read sequences can be found at the following NCBI BioProject ID: PRJNA727483. The ONT and Hi-C data, including the mtDNA genome, can be found at the following NCBI BioProject ID: PRJNA777104. The metagenome has been submitted to NCBI at the following BioProject: PRJNA906625. The methylation data can be found at the following BioProject: PRJNA991497.
