# Supplementary material for: The hologenome of Daphnia magna reveals possible DNA methylation and microbiome-mediated evolution of the host genome
Source: Nucleic Acids Res. 2023 Aug 28;51(18):9785–803. doi: 10.1093/nar/gkad685 (PMC10570034; doi:10.1093/nar/gkad685)

The hologenome of *Daphnia magna* reveals possible DNA methylation and microbiome-mediated evolution of the host genome

Anurag Chaturvedi[1], Xiaojing Li[1], Vignesh Dhandapani[1], Hollie Marshall[1,2], Stephen Kissane[1], Maria Cuenca-Cambronero[1,3], Giovanni Asole[4], Ferriol Calvet[4], Marina Ruiz-Romero[4], Paolo Marangio[4], Roderic Guigó[4], Daria Rago[1], Leda Mirbahai[5], Niamh Eastwood[1], John K. Colbourne[1], Jiarui Zhou[1], Eamonn Mallon[2], Luisa Orsini[1,7*]

[1]Environmental Genomics Group, School of Biosciences, and Institute for Interdisciplinary Data Science and AI, the University of Birmingham, Birmingham, B15 2TT, UK
[2]Department of Genetics and Genome Biology, the University of Leicester, Leicester, LE1 7RH, UK
[3] Aquatic Ecology Group, University of Vic - Central University of Catalonia, 08500 Vic, Spain.
[4] Centre for Genomic Regulation (CRG), The Barcelona Institute for Science and Technology (BIST), Barcelona, Catalonia, Spain
[5]Warwick Medical School, University of Warwick, Coventry, CV4 7AL, UK
[6]The Alan Turing Institute, British Library, London NW1 2DB, UK

* To whom correspondence should be addressed. Tel:+4401214145894; Email: l.orsini@bham.ac.uk

The authors wish it to be known that, in their opinion, the first 4 authors should be regarded as joint First Authors

Table S1. Morphological landmarks for *Daphnia magna* developmental stages. Nine developmental stages, corresponding to 9 morphological landmarks were used to generate RNA Seq data on a female and a male induced clone of *D. magna* from the commercial strain IRCHA 5. The stage number, the developmental stage in hours from time zero and the corresponding morphological landmark are shown.

Stage number	Developmental stage (hours)	Morphological landmark
1	0-1	Uniform, round
2	5-6	Uniform, with membrane (post gastrulation)
3	15	Dents appear in the outline, asymmetric depigmentation
4	20	Second antenna becomes defined, no limbs visible
5	26	Second antenna and rst two limbs are visible and distinct
6	34-35	First moult over, embryo pear-shaped, antenna do not reach 1st limb
7	41	Ocellum pigmented (red), antenna to rst limb
8	46	Antenna to 2nd limb, abdominal movements, backshield to 4th limb
9	51	Immediately post 2nd moult, eyes still separate

Table S2. Predicted genes in *Daphnia magna*. Tab 1) gene name (geneID) and scaffold position of the total number of genes identified by the Funannotate pipeline, including start and end codon, and directional information. Information is provided when a gene is supported by the predictors in the Funannotate pipeline (PASA, GeneMark, SNAP and Augustus), the gene modelers (geneID, CLASS, Stringtie and Scripture), Pfam, orthology to at least one other species in a list of 50, and orthology to at least one other *Daphnia* species. Genes with paralogs are also listed. For each gene the total number of independent supporting pieces of evidence is shown. For each gene, the protein length, KO terms (when known) and GO ontology are listed; Tab 2) *Daphnia magna* genes with orthology to other 50 species; Tab 3) *Daphnia magna* genes with orthology to other *Daphnia* species, including *D. galeata*, *D. obtusa*, *D. pulicaria* and *D. pulex*.

See Chaturvedi_etal_Table S2

Table S3. Sequencing data overview. Sequencing data statistics for Illumina and Oxford Nanopore (Nano) technologies generated libraries are shown for the LRV0_1 isolate. Number of reads, mean read length (bp) and total bases sequenced (bp) are shown. Four Illumina libraries and four Nanopore libraries (different insert lengths) were used.

LRV0_1	Number of reads	Mean read length (bp)	Total bases (bp)
Illumina			
Dmagna LRV0_1 - 1	14,442,306	250	3,625,018,806
Dmagna LRV0_1 - 2	14,330,600	250	3,596,980,600
Dmagna LRV0_1 - 3	14,125,140	250	3,545,410,140
Dmagna LRV0_1 - 4	63,125,048	150	9,365,526,712
**Total**	**106,023,094**		**20,132,936,258**
Nanopore			
Nano:8Kb	1,281,741	3,467	4,444,672,781
Nano: 48Kb	2,738,835	5,624	15,403,075,193
Nano:15Kb	2,904,177	4,645	13,489,977,990
Nano:15Kb	3,626,940	4,209	15,268,518,728
**Total**	**10,551,693**		**48,606,244,692**

Table S4. GenomeScope profiling. *Daphnia magna* LRV0_1 genome profiling from short reads, using GenomeScope (Vurture *et al*. 2017). Heterozygosity, haploidy, genome repeat length, and genome unique length are estimated from unprocessed short reads. The estimates are based on k-mer profiles, and the fit and error rate of these estimates are provided. The estimates in this table are complemented by the plot in Figure S7.

	**min**	**max**
Heterozygosity	0.85%	0.86%
Genome Haploid Length (bp)	133,054,791	133,206,470
Genome Repeat Length (bp)	36,456,744	36,498,304
Genome Unique Length (bp)	96,598,047	96,708,166
Model Fit	95.23%	99.14%
Read Error Rate	0.84%	0.84%

Table S5. ***Daphnia magna* assembly mapping onto linkage groups.** The linkage groups identified with the Hi-C proximity ligation technology and refined with short Illumina reads and Oxford Nanopore long reads are mapped onto the *D. magna* linkage map (Dukic *et al.* 2016). For each linkage ground (LG) identified in this study, the corresponding scaffold identified by Dukic (Dukic *et al.* 2016) is shown. The total number of uniquely mapped SNPs from the previously published linkage map and the length in bp for each linkage group forming a chromosome are shown.

**Linkage Group (Hi-C)**	**Scaffold ID (Dukic *et al.*, 2016)**	**No. of unique mapped SNPs**	**Chromosome length (bp)**
LG1	scaffold_2	427	14,684,576
LG2	scaffold_1	678	16,263,983
LG3	scaffold_3	293	13,877,452
LG4	scaffold_6	432	11,780,122
LG5	scaffold_4	383	13,735,328
LG6	scaffold_10	371	8,834,892
LG7	scaffold_5	351	11,903,208
LG8	scaffold_9	336	10,143,858
LG9	scaffold_7	308	10,938,020
LG10	scaffold_8	226	10,482,896

Table S6. Transposable elements. The transposable elements (TE) in the *Daphnia magna* LRV0_1. The type, number, and length of TEs are shown. The proportion of TE classes (%) in the genome is shown.

Type of TE	No of TE	Length (bp)	Proportion of TE in the genome
**Retroelements**	29421	16915936	13.01
SINEs	1356	286755	0.22
Penelope	92	53335	0.04
LINEs	3806	2098776	1.61
CRE/SLACS	0	0	0
L2/CR1/Rex	1296	322954	0.25
R1/LOA/Jockey	452	486424	0.37
R2/R4/NeSL	459	632507	0.49
RTE/Bov-B	252	19719	0.02
L1/CIN4	512	38847	0.03
LTR elements	24259	3065949	11.18
BEL/Pao	2083	3065949	2.36
Ty1/Copia	912	631039	0.49
Gypsy/DIRS1	20425	10562033	8.13
Retroviral	460	32014	0.02
**DNA transposons**	8466	2917392	2.24
hobo-Activator	3247	893472	0.69
Tc1-IS630-Pogo	1138	287750	0.22
En-Spm	0	0	0
MuDR-IS905	0	0	0
PiggyBac	190	1911	0.01
Tourist/Harbinger	428	59867	0.05
Other (Mirage, P-element,	458	268564	0.21
**Rolling-circles**	396	211553	0.16
**Unclassified**	42634	17181608	13.22

**Total interspersed repeats**		37014936	28.48
**Small RNA**	2452	920395	0.71
**Satellites**	494	159544	0.12
**Simple repeats**	45361	1570967	1.21
**Low complexity**	11219	513092	0.39

Table S7. Development genes in LRV0_1. List of putative novel genes identified within the developmental RNA-Seq data. The table includes the following information: gene name (geneID); the number of modelers supporting the gene identification among CLASS, Scripture, Stringtie and geneid (modeler); the location of the gene on the LRV0_1 assembly, including strand orientation, start and end codon; and correspondent location on the previously published genes set (Orsini, *et al.* 2016).

See Chaturvedi_etal_Table S7

Table S8. Protein coding genes shared between the *D. magna* LRV0_1 isolate and the published reference gene set. The following are shown: gene ID in the LRV0_1 isolate (LRV0_1 geneID) as predicted by the Funannotate pipeline; gene supported by multiple pieces of evidence (evidence); corresponding gene in the reference gene set (published gene set geneID); percentage of identical nucleotides shared between LRV0_1 and the published reference (% identity) and the gene coverage in LRV0_1 (coverage) are also shown. KO terms identified in the LRV0_1 and the reference gene set. A KO term identified in both is marked with 1. Also shown are the scaffold location, start and end codon, and strand orientation. In the last column of this spreadsheet, the LRV0_1 geneID and the correspondent geneID on the reference gene set are shown.

See Chaturvedi_etal_Table S8

Table S9. Tukey HSD post hoc test on codon degeneracy. Results are shown after correcting for multiple testing for differences between codon degeneracy levels at housekeeping and all other genes. Significant contrasts are in bold phase.

Contrast	Estimate	SE	z.ratio	P-value
degeneracy0 Housekeeping - degeneracy2 Housekeeping	-0.09597	0.0285	-3.368	**0.0173**
degeneracy0 Housekeeping - degeneracy3 Housekeeping	0.44549	0.1007	4.423	**0.0003**
degeneracy0 Housekeeping - degeneracy4 Housekeeping	0.43331	0.0281	15.397	**<.0001**
degeneracy0 Housekeeping - degeneracy0 Other	2.73862	0.0512	53.501	**<.0001**
degeneracy0 Housekeeping - degeneracy2 Other	2.70496	0.0927	29.167	**<.0001**
degeneracy0 Housekeeping - degeneracy3 Other	3.26181	0.3539	9.216	**<.0001**
degeneracy0 Housekeeping - degeneracy4 Other	2.73324	0.0728	37.567	**<.0001**
degeneracy2 Housekeeping - degeneracy3 Housekeeping	0.54146	0.1029	5.262	**<.0001**
degeneracy2 Housekeeping - degeneracy4 Housekeeping	0.52928	0.0352	15.045	**<.0001**
degeneracy2 Housekeeping - degeneracy0 Other	2.83459	0.0554	51.194	**<.0001**
degeneracy2 Housekeeping - degeneracy2 Other	2.80093	0.0951	29.449	**<.0001**
degeneracy2 Housekeeping - degeneracy3 Other	3.35779	0.3546	9.47	**<.0001**
degeneracy2 Housekeeping - degeneracy4 Other	2.82921	0.0758	37.346	**<.0001**
degeneracy3 Housekeeping - degeneracy4 Housekeeping	-0.01218	0.1028	-0.118	1
degeneracy3 Housekeeping - degeneracy0 Other	2.29313	0.1113	20.595	**<.0001**
degeneracy3 Housekeeping - degeneracy2 Other	2.25947	0.1356	16.667	**<.0001**
degeneracy3 Housekeeping - degeneracy3 Other	2.81633	0.3675	7.664	**<.0001**
degeneracy3 Housekeeping - degeneracy4 Other	2.28775	0.1228	18.636	**<.0001**
degeneracy4 Housekeeping - degeneracy0 Other	2.30531	0.0552	41.771	**<.0001**
degeneracy4 Housekeeping - degeneracy2 Other	2.27165	0.095	23.911	**<.0001**
degeneracy4 Housekeeping - degeneracy3 Other	2.8285	0.3545	7.978	**<.0001**
degeneracy4 Housekeeping - degeneracy4 Other	2.29993	0.0756	30.412	**<.0001**
degeneracy0 Other - degeneracy2 Other	-0.03365	0.1042	-0.323	1
degeneracy0 Other - degeneracy3 Other	0.5232	0.3571	1.465	0.8261
degeneracy0 Other - degeneracy4 Other	-0.00538	0.0869	-0.062	1
degeneracy2 Other - degeneracy3 Other	0.55685	0.3654	1.524	0.7947

degeneracy2 Other - degeneracy4 Other	0.02828	0.1163	0.243	1
degeneracy3 Other - degeneracy4 Other	-0.52858	0.3608	-1.465	0.8262

Table S10. DNA methylation per coding region. Dunn postdoc test with Benjamini-Hochberg adjusted and non-adjusted P-values for DNA methylation levels between differing exons, with any exon above 6 labeled as ‘n’.

Exons	Z	P-value	Adjusted P-value
1 - 2	-20.4633823	4.565845e-93	1.369754e-92
1 - 3	-22.809379	3.70E-115	1.85E-114
2 - 3	-3.4394217	5.83E-04	6.25E-04
1 - 4	-10.494558	9.15E-26	1.53E-25
2 - 4	7.8912426	2.99E-15	4.08E-15
3 - 4	10.6750598	1.33E-26	2.50E-26
1 - 5	0.9052047	3.65E-01	3.65E-01
2 - 5	18.4088019	1.12E-75	2.79E-75
3 - 5	20.6639013	7.32E-95	2.75E-94
4 - 5	10.0459873	9.57E-24	1.44E-23
1 - n	6.911405	4.80E-12	6.00E-12
2 - n	31.3982781	2.14E-216	1.60E-215
3 - n	33.071727	7.58E-240	1.14E-238
4 - n	18.0188101	1.39E-72	2.97E-72
5 - n	4.5363518	5.72E-06	6.60E-06

Table 11. Enriched GO terms. Enriched GO terms are shown for highly methylated genes and genes with no methylation. GO term ID, predicted function, frequency, uniqueness and dispensability of the GO terms are shown for both types of genes. Dispensability is a measure used to identify genes that can be inactivated without affecting an organism's viability.

See Chaturvedi et al. Table S11

Table S12 Structural annotation of the draft metagenomes. Structural annotation of the draft bacterial genomes listed in Table 2 of the main text. For each taxon, the number of contigs, the genome length (bp), the number of ribosomal RNA (rRNA), transfer RNA (tRNA), transfer-messenger RNA (tmRNA), and protein coding sequence (CDS) are shown.

Bacterial taxon	No of	Genome	No of	No of	No of	No fo
Cytophagales (order)	493	5,938,772	2	36	2	4957
Burkholderiales	203	5,683,207	3	65	1	4827
Burkholderiales	1287	5,428,086	1	45	0	5514
Cytophagales (order)	258	4,966,502	2	39	1	4208
Burkholderiales	1258	3,781,125	1	37	0	3978
Deltaproteobacteria	1581	2,510,294	2	17	1	2790
Bacteroidota (phylum)	142	2,723,662	0	32	1	2403
Bacteria (kingdom)	89	735,906	2	35	1	736

Table S13 *Daphnia magna* microbiota composition. Gut microbiota composition expressed in read counts and relative abundance (%) for phylum (tab 1), class (tab 2), order (tab 3), family (tab 4) and genus (tab 5). The tab 'comparison' provides a summary at glance of the microbial community composition in the *D. magna* gut, the growth medium (borehole water) and the algal feed. This Table supports Figure 4 in the main text.

See Chaturvedi_etal. Table S13

Table S14 AMR and VF. Antimicrobial resistant genes (AMR) and virulence factors (VF) identified in the metagenome of *Daphnia magna*. Tab 1 is the legend to Tab AMR and VF. This table supports Figure 5 in the main text.

See Chaturvedi_etal. Table S14

Figure S1 ***Daphnia magna*** **hybrid assembly pipeline.** The wtdbg2 pipeline was used for the LRV0_1 isolate assembly, including long reads generated with Oxford Nanopore Technologies (ONT), and short Illumina reads. The final assembly was anchored onto the chromosomal level assembly obtained from the proximity ligation Hi-C data.

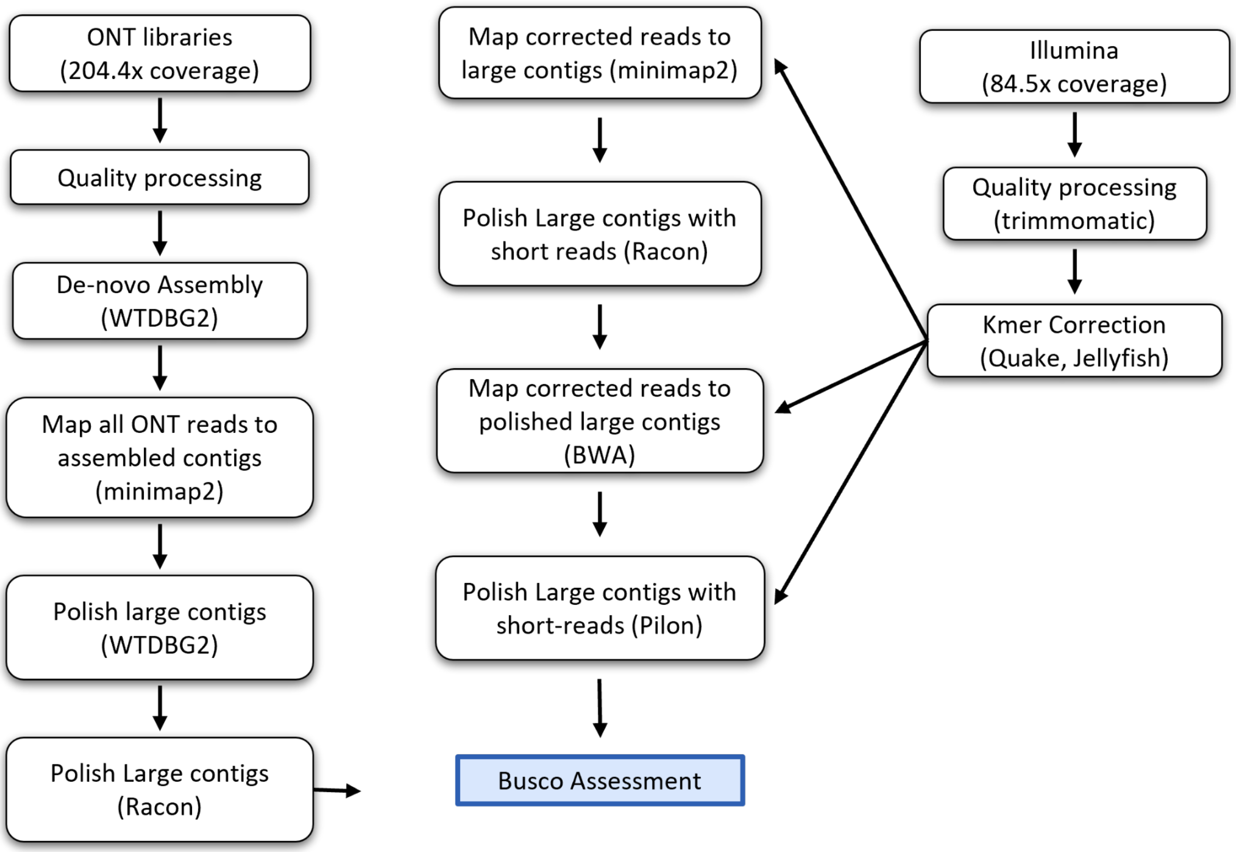

Figure S2 Decision tree. Genes prediction and functional annotation decision tree used to define the gene set in the *Daphnia magna* LRV0_1 isolate, supported by multiple pieces of evidence.

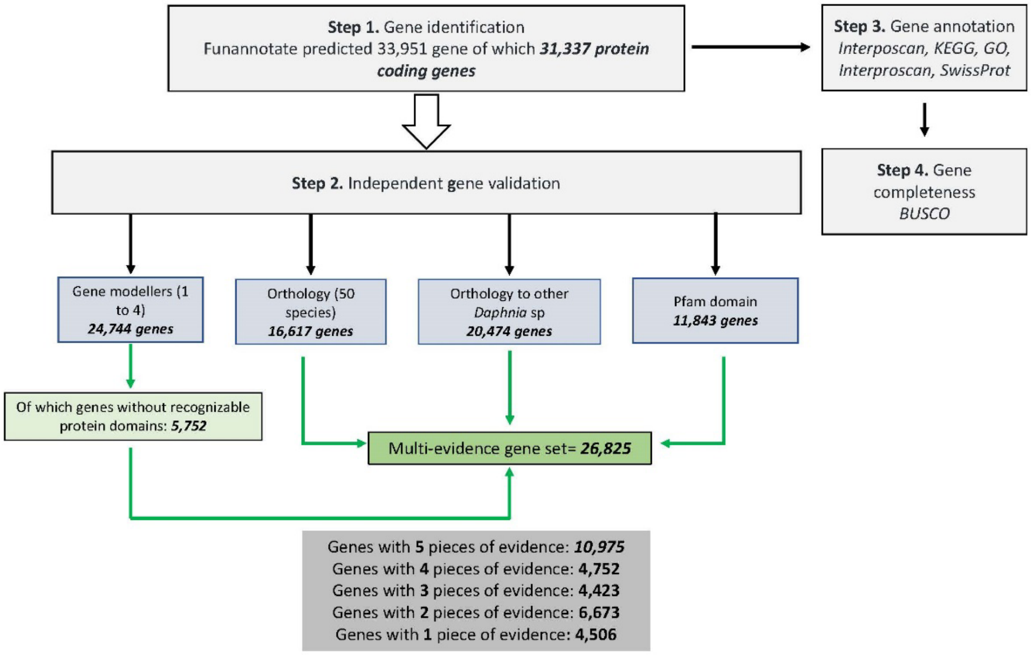

Figure S3. Eukaryotic Genome Annotation Pipeline Funannotate. Schematic diagram of the Funannotate pipeline, a genome prediction tool, to generate gene prediction models trained on RNAseq data. In our case the mRNA data were obtained from the exposure of LRV0_1 to a suite of 12 environmental conditions described in methods.

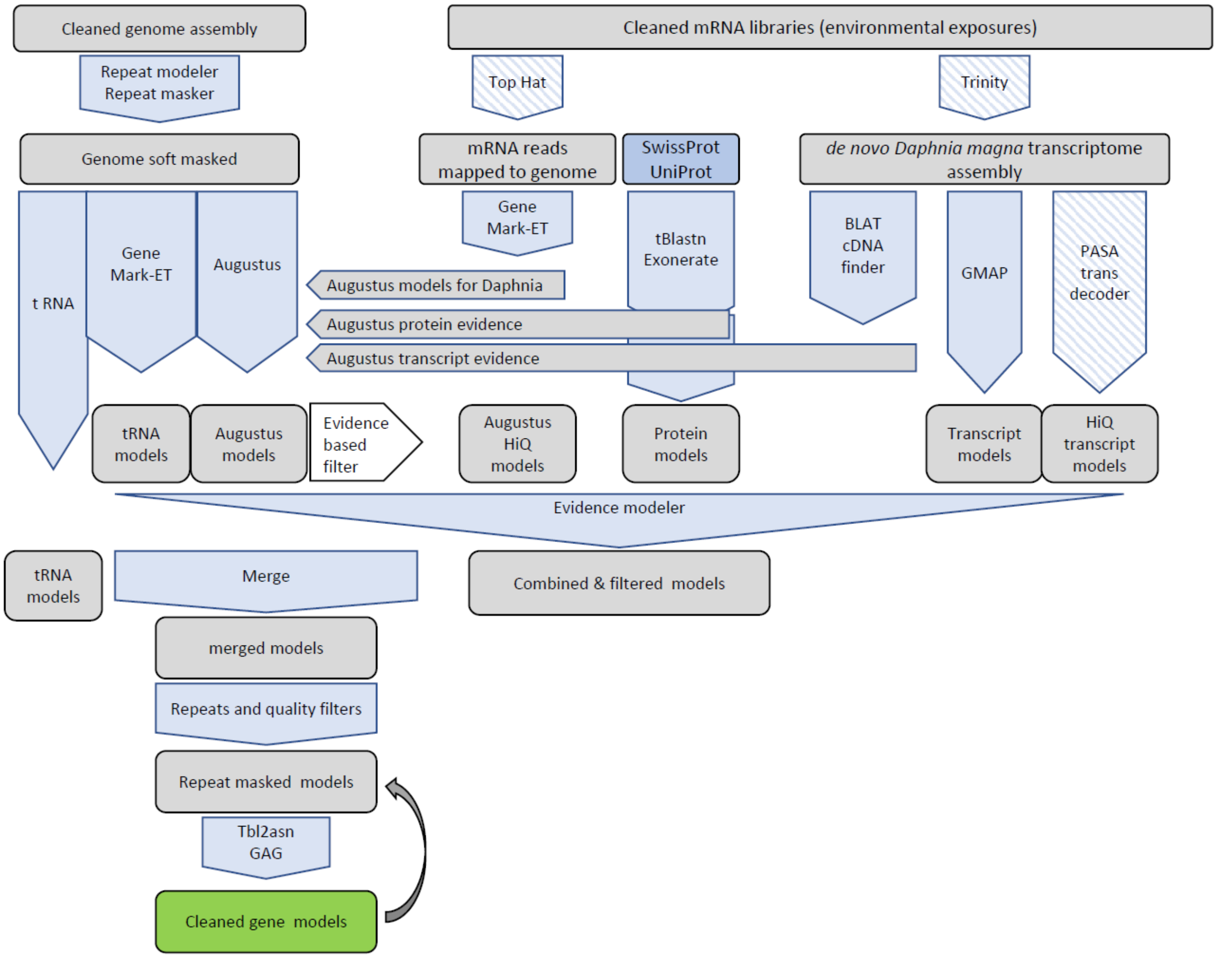

Figure S4. Venn diagram of predicted genes by four gene modelers. Genes validated by up to four gene modelers (Class, GeneID, Stringtie and Scripture) using independent RNAseq data obtained from nine morphological landmarks listed in Table S1. The overlap of gene predictions among the four gene modelers is displayed.

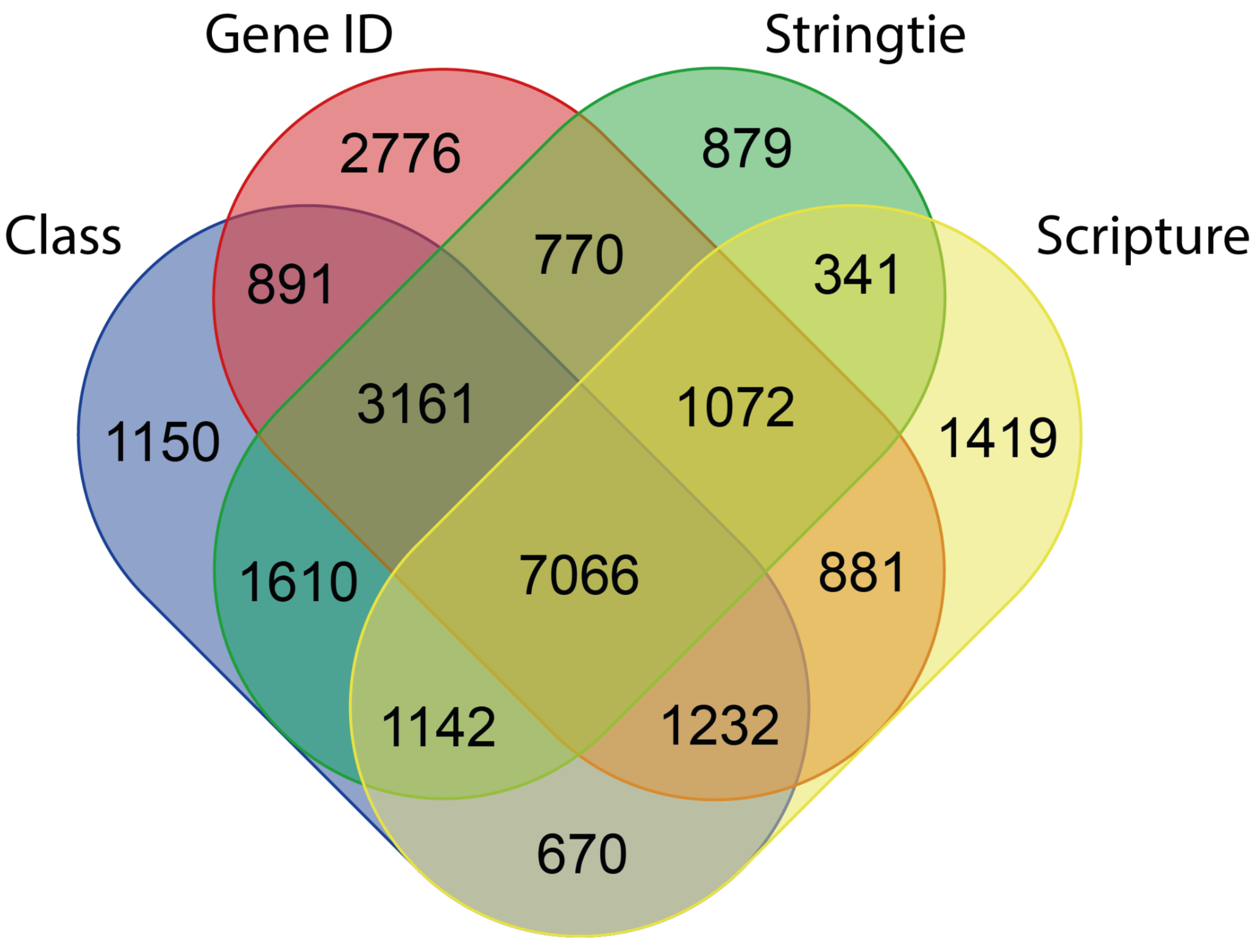

Figure S5 Comparative performance of metagenome annotation based on five binning algorithms. Five binning algorithms were applied to deconvolute the metagenome of *D. magna*, i.e., Proximeta, MetaBat2, MaxBin2, DASTool and BinSanity (LC mode). The (A) completeness and (B) purity of the draft genomes obtained with these 5 algorithms (color coded) were subsequently evaluated with CheckM.

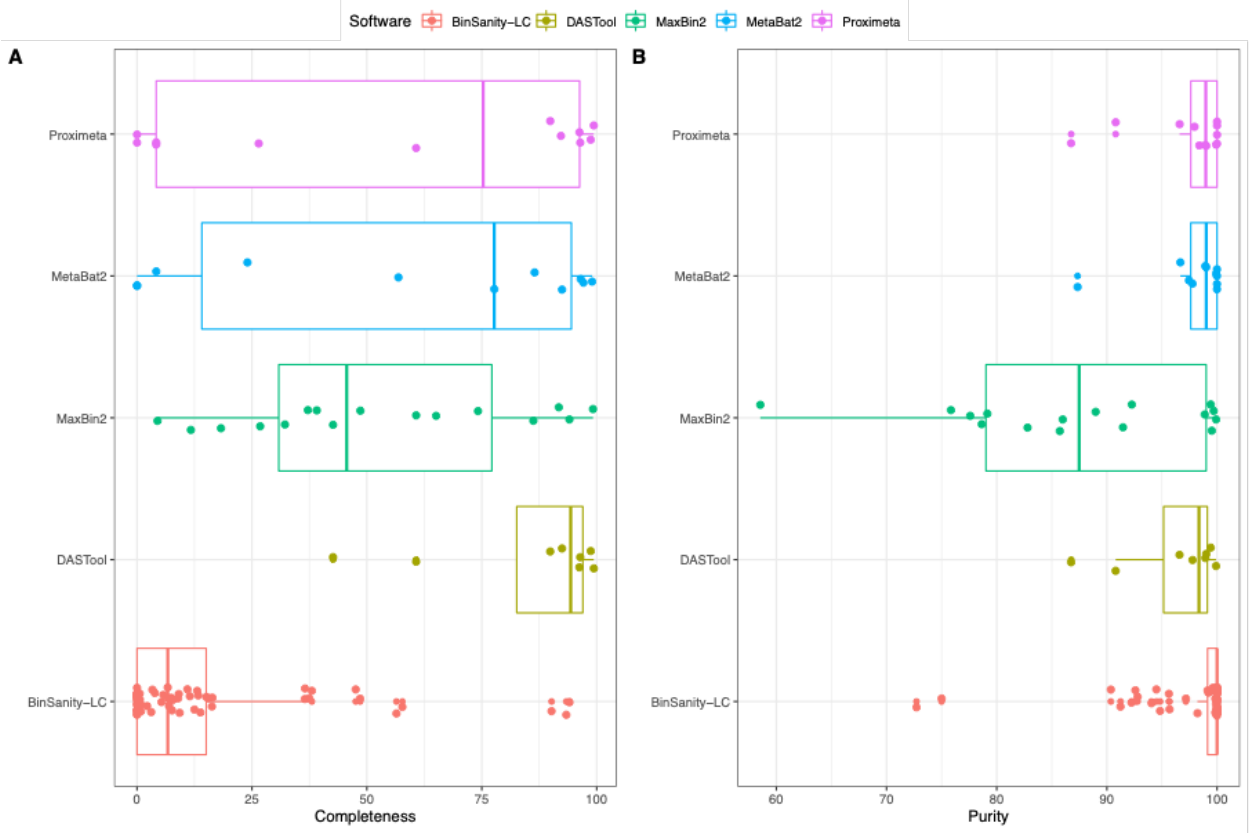

Figure S6. Chromosomal-level genome assembly. Post scaffolding chromosomal-level heat map, arranged by chromosome size and including the SNP markers used to build the *Daphnia magna* linkage map. The link intensity is shown in logarithmic scale (log_{10}).

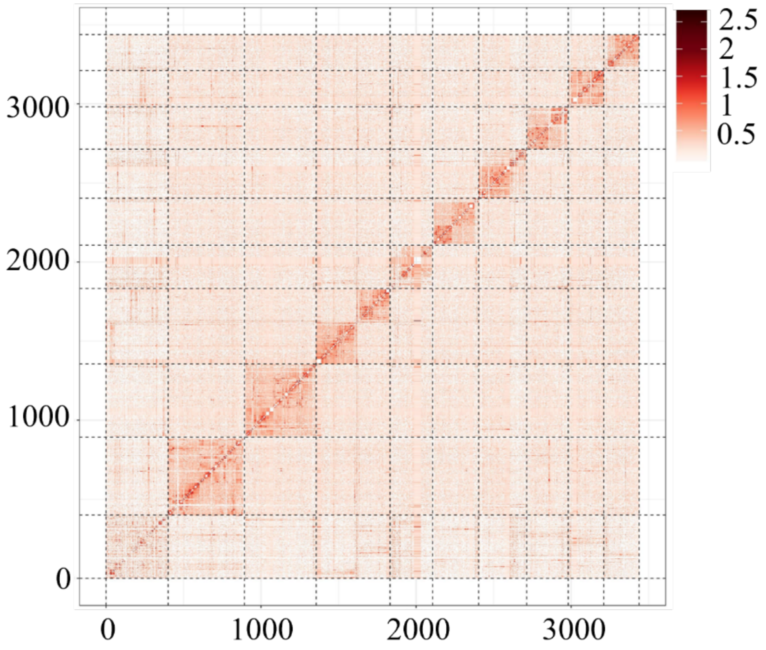

Figure S7. GenomeScope profile of the *D. magna* LRV0_1 genome. The plot shows the observed genome profile from Illumina short reads and the fit to the estimated genome length based on k-mer (full model). Unique sequences, error rate and k-mer peaks are also shown. The plot in this figure complements estimates presented in Table S4.

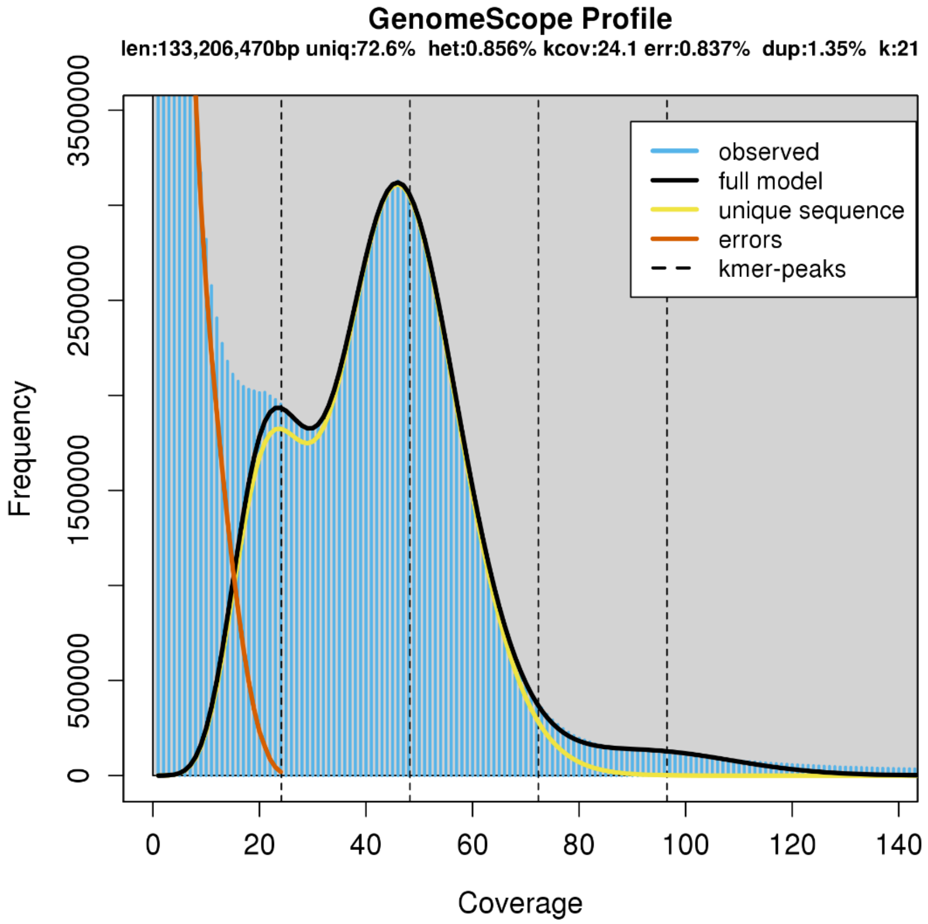

Figure S8 DNA methylation patterns. (A) Mean DNA methylation levels per exon within genes. Error bars represent 95% confidence intervals. (B) Gene expression levels of all genes binned by DNA methylation level (n = 18,817). The blue line represents a linear regression with the gray shaded area showing 95% confidence intervals. (C) DNA methylation levels of bins across all ATAC peaks across the genome, including 1Kb upstream and downstream of all peaks. (D) DNA methylation levels of bins across ATAC peaks across genes, including 1Kb upstream and downstream of all peaks.

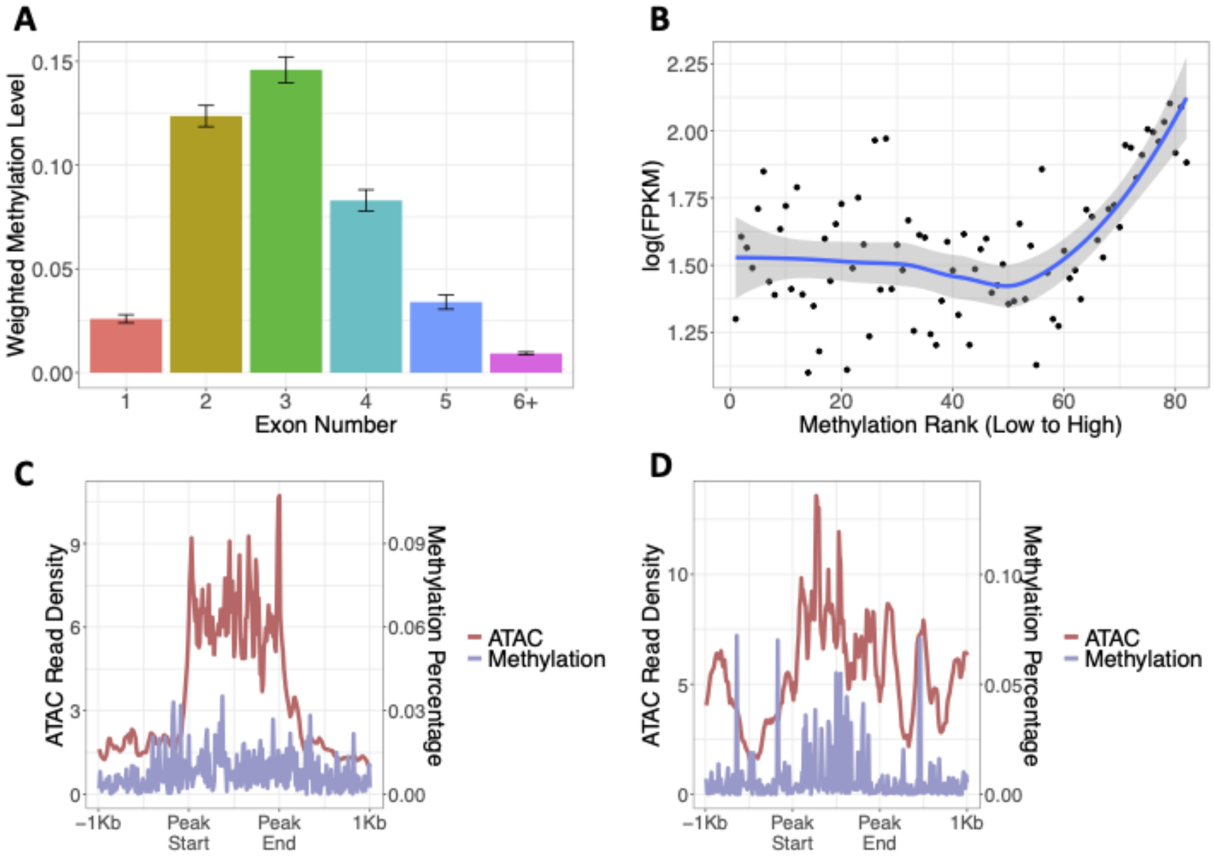

Figure S9 DNA methylation patterns. (A) Mean DNA methylation levels (weighted methylation) across genomic regions. Error bars represent 95% confidence intervals. (B) Barplot of the DNA methylation levels of various transposable elements (TE), error bars represent 95% confidence intervals of the mean, the red numbers represent the *n* of each TE category.

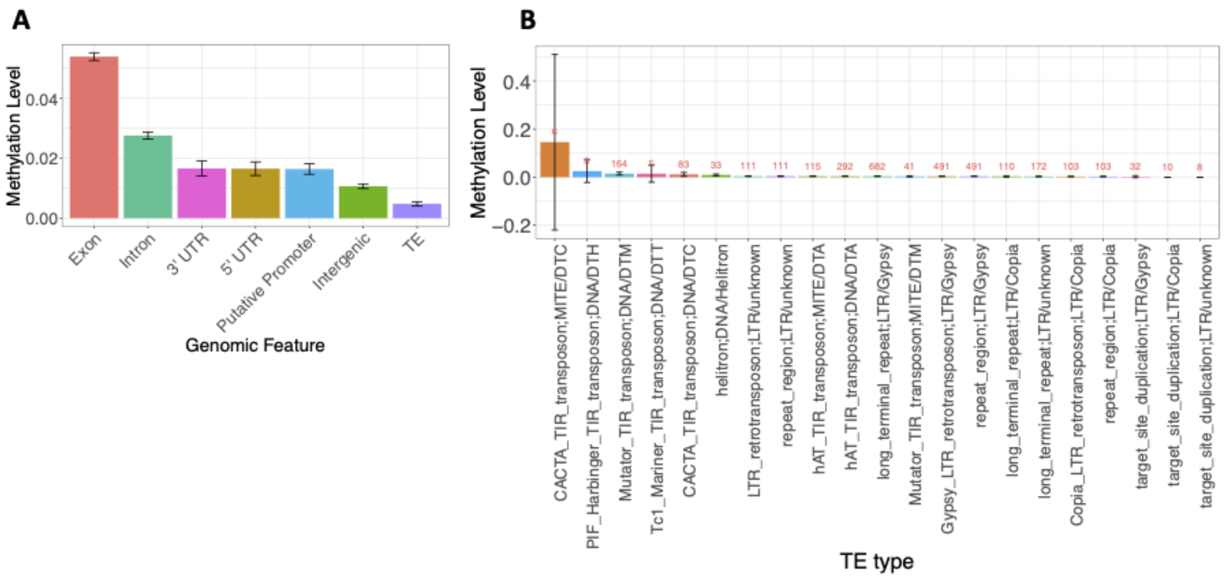

Figure S10 DNA methylation function. Schematic representation of two alternative theories explaining the function of gene-body DNA methylation: a) DNA methylation works as a stabilizing factor; b) DNA methylation is a mutagen, providing a genetic substrate for selection.

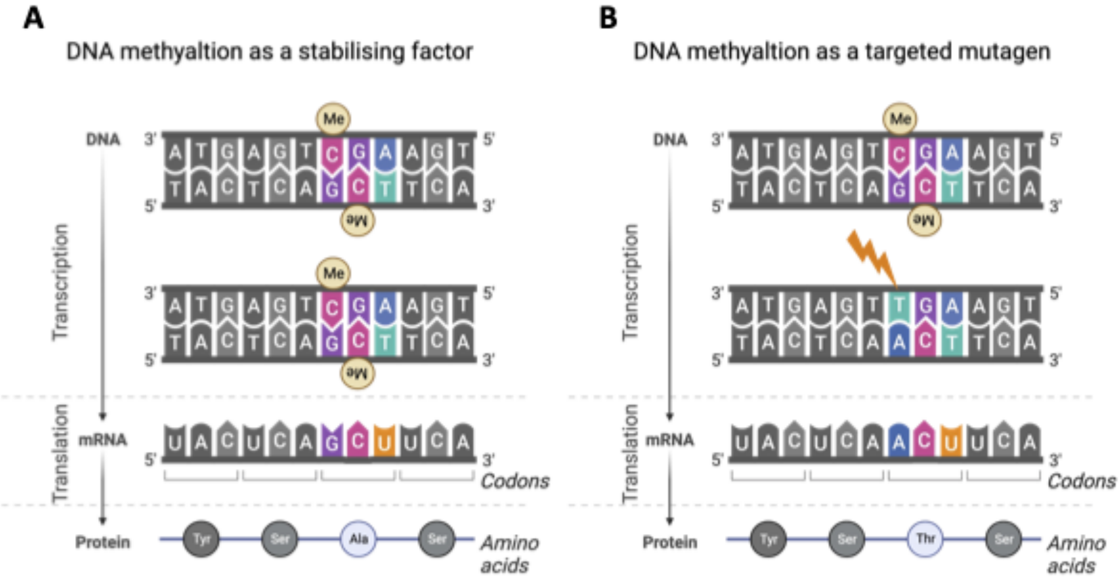

Supplement: gkad685_Supplemental_Files [file gkad685_supplemental_files.zip › Chaturvedi_et_al_supplementary_infoNAR R3.pdf]
